# Assessment of ovarian dysfunction induced by environmental toxins: a systematic review

**DOI:** 10.3389/fpubh.2025.1575418

**Published:** 2025-07-30

**Authors:** Lu An, Yali Huang, Yunkai Wang, Shuting Shen, Xuexing Luo, Xiaoyan Liang, Liming Lu, Chunzhi Tang, Jinglin Lin, Ting Su, Meiqi Zhan, Dongying Wang, Jue Wang, Xin Lai, Yu Li

**Affiliations:** ^1^Faculty of Chinese Medicine, Macau University of Science and Technology, Macao, Macao SAR, China; ^2^Faculty of Humanities and Arts, Macau University of Science and Technology, Macao, Macao SAR, China; ^3^Department of Reproductive Center, The Sixth Affiliated Hospital, Sun Yat-sen University, Guangzhou, China; ^4^Biomedical Innovation Center, The Sixth Affiliated Hospital, Sun Yat-sen University, Guangzhou, China; ^5^Medical College of Acu-Moxi and Rehabilitation, Guangzhou University of Chinese Medicine, Guangzhou, China; ^6^Department of Traditional Chinese Medicine, The Sixth Affiliated Hospital, Sun Yat-sen University, Guangzhou, China; ^7^State Key Laboratory of Quality Research in Chinese Medicines, Macau University of Science and Technology, Macao, Macao SAR, China

**Keywords:** ovary, ovarian function, ovarian reserve, environmental pollutants, persistent organic pollutants, air pollutants, heavy metal

## Abstract

**Objective:**

This study systematically assess the potential impact of various environmental pollutants as chemical, airborne, and heavy metal on ovarian function in women, focusing on ovarian reserve such as anti-Müllerian hormone (AMH) and antral follicle count (AFC) as well as hormone levels like follicle-stimulating hormone (FSH) and estradiol (E2). By reviewing epidemiological evidence, this research aims to elucidate the reproductive toxicity of these pollutants and provide scientific support for public health policy to protect reproductive health in women of childbearing age.

**Methods:**

Following the PRISMA-P guidelines, a comprehensive search was conducted in PubMed, EMBASE, Cochrane Library, and Web of Science databases to include all relevant studies up to July 30, 2024. The Newcastle-Ottawa Scale (NOS) and the Grading of Recommendations Assessment, Development and Evaluation (GRADE) approach were used to assess study quality.

**Results:**

This study ultimately included 40 cohort study reports derived from 33 distinct studies that analyzed the effects of 20 pollutant types on ovarian function. Results indicate that pollutants, such as perfluoroalkyl and polyfluoroalkyl substances (PFAS), phthalates (PAEs), triclosan, Polychlorinated Biphenyls (PCBs), PM2.5, and SO_X_, have a significantly negative impact on ovarian function, especially among younger women (<35 years). Long-term exposure to particulate matter (PM)2.5 and PM10 is associated with a substantial decrease in ovarian reserve, while heavy metals (e.g., lead and cadmium) also demonstrate reproductive toxicity. However, these conclusions require validation due to both methodological limitations in the original studies (e.g., heterogeneous exposure assessments and residual confounding) and challenges in evidence synthesis (e.g., inconsistent outcome measures across cohorts), highlighting the need for further research to address these constraints.

**Conclusion:**

This review underscores that specific pollutants (e.g., PCBs, PFAS, PM) pose substantial risks to reproductive health in women of childbearing age, particularly in highly polluted environments. The findings underscore the importance of regular ovarian health monitoring, especially for women at higher risk due to occupational or environmental factors.

**Systematic review registration:**

PROSPERO CRD42024567744 (accessible at https://www.crd.york.ac.uk/prospero/display_record.php?ID=CRD42024567744).

## Introduction

Environmental pollutants pose significant global public health risks due to their increasing types and concentrations in in air, water, and soil driven by industrialization, urbanization, and agricultural modernization (1). Numerous studies have shown that air pollutants, such as particulate matter (PM) (2), ozone (O_3_), sulfur dioxide (SO_2_), and nitrogen oxides (NO_X_) (3), are significantly associated with respiratory diseases (4), cardiovascular diseases (5, 6), and neurological disorders (7, 8). For instance, long-term exposure to air pollution can increase the risk of atherosclerosis by inducing oxidative stress and inflammatory responses (9–11). Additionally, chemical pollutants including phthalates (PAEs) (12, 13) and bisphenol A (BPA) (14), act as endocrine disruptors (15), causing hormonal imbalances that can lead to diseases such as diabetes (16), obesity, and cancer (17–19). Persistent organic pollutants (POPs), which resist degradation (20), and heavy metals like lead (Pb), mercury (Hg), and cadmium (Cd) accumulate through the food chain, and prolonged exposure can cause kidney damage (21, 22), neurological disorders (23), and osteoporosis (24).

**Figure 1:**
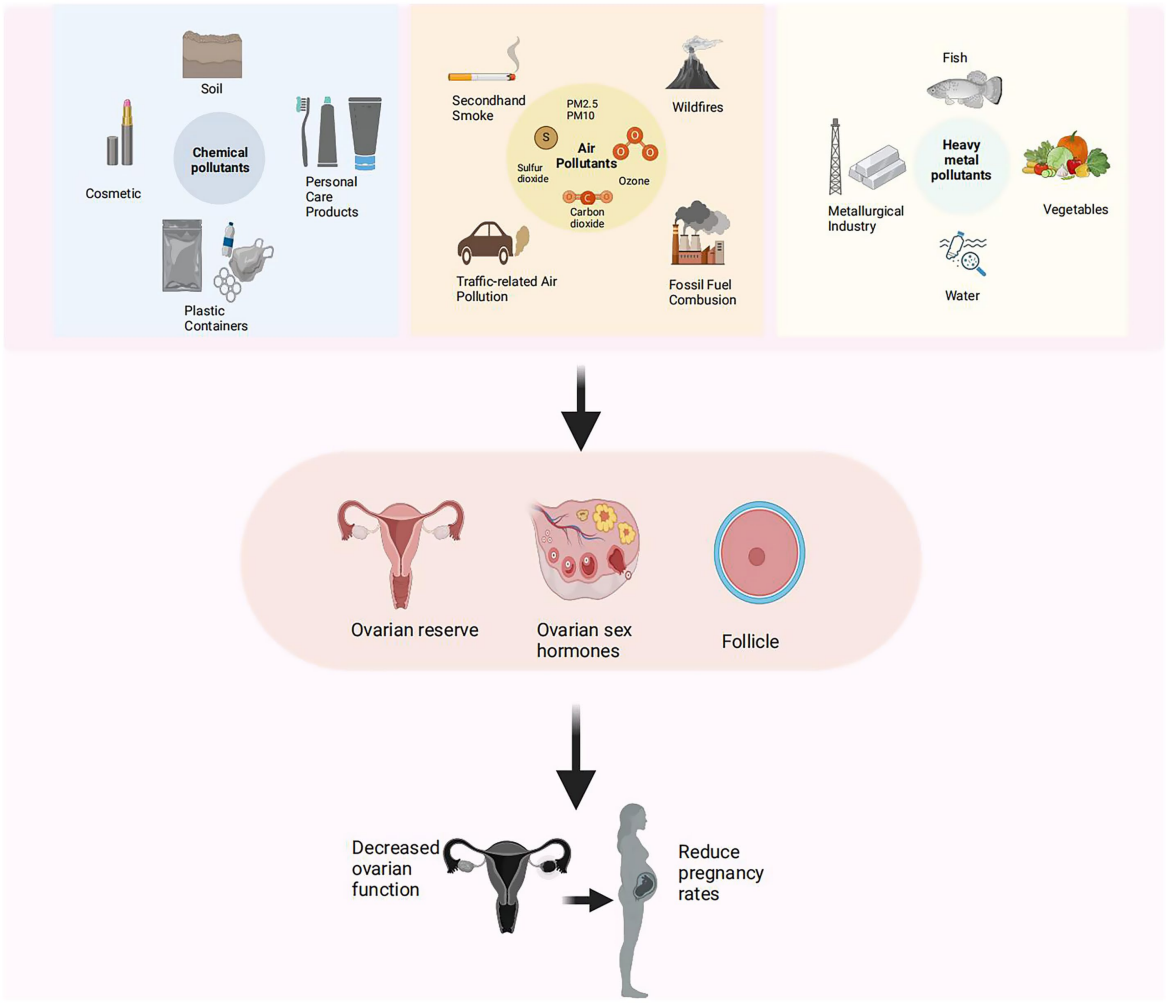
Effects of environmental pollutants and heavy metals on ovarian reserve and fertility. Schematic diagram illustrating sources of environmental pollutants (left), including chemical, airborne, and heavy metal contaminants, and their associations with reduced ovarian reserve, hormonal disruption, and lower pregnancy rates (right).

With the growing awareness of the harmful effects of environmental pollutants, increasing studies have begun to explore their impact on reproductive health, particularly ovarian function in women (25–27). The ovary, essential for oocyte production (28, 29) and sex hormone secretion (30), regulates reproductive functions and contributes to overall health and metabolism balance (31, 32). Ovarian dysfunction, linked to menstrual irregularities, infertility, premature ovarian failure, and increased risks of cardiovascular disease and osteoporosis (33–35), can be exacerbated by pollutants through mechanisms such as inflammation, oxidative stress, and endocrine disruption (36) (Figure 1). Environmental pollutants are classified as persistent and non-persistent types (37). Persistent pollutants [e.g., Polychlorinated Biphenyls (PCBs), Per-and Polyfluoroalkyl Substances (PFAS), dioxins], have stable chemical structures, enabling them remain in the environment for years or even decades (38, 39) and potentially disrupt ovarian reserve and oocyte quality through endocrine disruption and DNA damage (40–42). Non-persistent pollutants, such as PAEs (43) and BPA (44), are rapidly metabolized and excreted by the body (45). However, due to their widespread presence in daily life (46, 47), chronic low-level exposure to these compounds may still disrupt hormonal balance and contribute to ovarian dysfunction (48, 49). Air pollutants like PM2.5, PM10, O_3_, and NO_x_ (50–52), can damage ovarian cells by inducing inflammation and oxidative stress (53, 54). As well as long-term exposure to heavy metals like Pb and Cd promotes ovarian cell apoptosis, gene mutations, and DNA damage (55), reducing ovarian reserve (56) and increasing the risk of infertility (57).

In the following sections, several common and significant pollutants are categorized, with a focus on their widespread use, primary exposure pathways, half-life, and specific impacts on ovarian function. Please refer to Table 1 for all abbreviations and their full names.

**Table 1:** List of abbreviations.

Abbreviations	Full name	Abbreviations	Full name
POPs	Persistent organic pollutants	PM2.5	Particulate matter 2.5
PFAS	Per-and polyfluoroalkyl substances	PM10	Particulate matter 10
PFCs	Perfluorochemicals	PAHs	Polycyclic aromatic hydrocarbons
PFOS	Perfluorooctane sulfonate	SO_X_	Sulfur oxides
n-PFOA	n-Perfluorooctanoic acid	SO_2_	Sulfur dioxide
Sm-PFOS	Sum of branched-chain perfluorooctane sulfonate	NO_X_	Nitrogen oxides
PAEs	Phthalates	NO_2_	Nitrogen dioxide
MEP	Mono-ethyl phthalate	O_3_	Ozone
MBzP	Mono-benzyl phthalate	CO	Carbon monoxide
DEHP	Di (2-ethylhexyl) phthalate	BTEX	Benzene, Toluene, Ethylbenzene, and Xylene
MiNP	Mono-iso-nonyl phthalate	Hg	Mercury
MiDP	Mono-iso-decyl phthalate	Cd	Cadmium
MiBP	Mono-iso-butyl phthalate	Pb	Lead
MBP/MnBP	Mono-n-butyl phthalate	Cr	Chromium
MEHP	Mono-(2-ethyl-hexyl)phthalate	Ba	Barium
MEOHP	Mono(2-ethyl-5-oxohexyl) phthalate	As	Arsenic
DBP	Di-n-butyl phthalate	E2	Estradiol
BPs	Benzophenones	P	Progesterone
OCPs	Organochlorine pesticides	T	Testosterone
HCB	Hexachlorobenzene	AFC	Antral follicle count
DDT	Dichloro-diphenyl-trichloroethane	AMH	Anti-Müllerian hormone
DDE	Dichlorodiphenyldichloroethylene	FSH	Follicle-stimulating hormone
PCBs	Polychlorinated biphenyls	INHB	Inhibin B
BPA	Bisphenol A	LH	Luteinizing hormone
MP	Methylparaben	FF	Follicular fluid
PP	Propylparaben	ER	Estrogen receptors
TCDD	2,3,7,8-tetrachlorodibenzo-p-dioxin	PCOS	Polycystic ovary syndrome
TCS	Triclosan	ART	Assisted reproductive technologies
PM	Particulate matter	IVF	In vitro fertilization
n3PUFA	n-3 polyunsaturated fatty acids	AI	Artificial intelligence
BMI	Body mass index		

Full terminology corresponding to abbreviated forms used throughout the text.

### Chemical pollutants

PFAS, PAEs, organochlorine pesticides (OCPs), PCBs, dioxin, parabens, and BPA are widespread in industry, agriculture, soil, and daily necessities due to their chemical stability. These substances enter the human body through drinking water, food, air and skin contact, accumulating over time and adversely affect ovarian function (58).

**PFAS** are a class of synthetic chemicals reknowned for their high stability, with half-lives exceeding half a century in the environment (39). They are widely used in waterproof, oil-repellent, and stain-resistant materials, as well as heat-resistant products, such as food packaging, non-stick cookware, and textiles (59). Exposure occurs primarily through drinking water, food, and inhalation, with PFAS persisting in the human body for 3 to 8.5 years (60). Studies indicated that PFAS exposure significantly impacts ovarian health by inducing excessive reactive oxygen species and triggering cell apoptosis through the intrinsic pathway (61). This oxidative stress impairs mitochondrial function and disrupts structural stability of the oocyte complex, ultimately leading to oocyte apoptosis and necrosis (62).

**PAEs** are common plasticizers widely used in plastic products, cosmetics, and cleaning products. Despite PAEs have a relatively short half-life (12 h) (63), accumulate with frequent exposure via ingestion, inhalation and skin contact (45, 64), can lead to long-term adverse effects on the human body. PAEs can enhance inflammatory gene expression in human ovaries while reducing ovarian cholesterol and steroid synthesis (65). Animal experiments revealed that exposure to di (2-ethylhexyl) phthalate (DEHP) in female lactating mice significant increase in the expression of DNA damage marker γH2AX in ovarian cells, intensified ovarian cell apoptosis, and notably inhibits proliferation of ovarian granulosa cells (66).

**OCPs,** like dichlorodiphenyltrichloroethane (DDT) and hexachlorobenzene (HCB), were once widely used in agriculture (67). Although they have been banned (68), they remain in the environment due to their stable physicochemical properties, at high and persistent concentrations, with half-lives in soil that can reach 20 to 30 years (69). OCPs can enter the human body through the food chain, especially through animal-derived foods including poultry, meat, eggs, milk, and in particular, animal fat (70). Once absorbed, OCPs can persist for 10 to 20 years, with the dichlorodiphenyldichloroethylene (DDE) component potentially remaining for a lifetime (71). OCPs disrupt hormone signaling pathways, exhibiting anti-estrogenic and anti-androgenic effects. By inhibiting aromatase activity and steroidogenic enzyme systems, they disrupt endocrine function and negatively impact gonadal health (71).

**PCBs** have been widely used in industrial applications such as printing inks, copying paper, coolants and insulating materials for electrical equipment, paints, and lubricants (72). Although they are now banned internationally, PCBs are still slowly and persistently released from old equipment and landfills into environmental air, soil, water, and sediments (72, 73), eventually contaminating the human food chain (74). Their long half-life in the human body, lasting decades, poses a prolonged health risk (75). PCBs disrupt the expression of crucial enzyme genes, such as CYP17, 3β-HSD, and CYP19 (76, 77), involved in steroid hormone biosynthesis, thereby negatively impacting the synthesis and secretion of ovarian hormones.

**Dioxins** are not produced intentionally and have no practical value, they are unintentional by-products of numerous industrial activities and all combustion processes (59, 78). These chemicals are highly persistent in the environment, particularly 2,3,7,8-Tetrachlorodibenzo-p-dioxin (TCDD), which has a half-life of 10 to 12 years in soil (79). Human exposure occurs primarily through dietary intake, especially from the consumption of meat, milk, eggs, fish, and related products (80), with a half-life in the human body ranging from 5 to 10 years (81). A retrospective cohort study revealed that each 10-fold increase in serum TCDD levels was associated with a 25% prolongation of time-to-pregnancy (TTP) and an approximate doubling of infertility risk (82). Animal studies showed that TCDD exposure increases granulosa cell apoptosis in growing follicles and disrupts follicular maturation (83), leading to a reduction in pre-antral and antral follicle numbers (84).

**Parabens**, widely used as preservatives in cosmetics, pharmaceuticals, and food products (85), are among the most prevalent ingredients in cosmetics and personal care products after water (86). Although their half-life in the body relatively short (a few hours to days) (87), exposure through skin absorption or ingestion can trigger oxidative stress responses in the body (88), leading to ovarian cell damage or apoptosis (89). These effects may ultimately impair fertility.

**BPA** is widely used in the production of plastic products, food cans, and thermal paper (90). The primary route of BPA exposure is ingestion of contaminated food (91). Although its half-life in the body is short (4–6 h) (92), continuous exposure from daily necessities results in long-term endocrine disruption (93). An *in vitro* study demonstrated that exposure to 100 μM BPA significantly inhibited cell proliferation and promoted autophagy in human granulosa cells (94). Studies in mice found that BPA impairs folliculogenesis, leading to cystic expansion of the follicles, a decrease in the number of granulosa cells within the follicles, and a corresponding decline in hormone levels associated with granulosa cells, including estradiol (E2), progesterone (P), and anti-Müllerian hormone (AMH) (95).

**Benzophenones (BPs)** are commonly found in the formulations of sunscreens, nail polishes, enamel paints, bath products, and skincare products, absorbed through skin and have a half-life of a few days (96). Hydroxylated forms such as benzophenone-3 (BP-3), benzophenone-1 (BP-1), and benzophenone-2 (BP-2) exhibit estrogenic and anti-androgenic activities in both *in vitro* and *in vivo* experiments (97, 98), thereby interfering with the reproductive system specifically by inhibiting the development of oocytes (98).

**Triclosan (TCS)** is an antimicrobial agent in personal care products such as soaps, toothpaste, and deodorants, as well as medical devices (99). TCS primarily absorbed through skin absorption and oral ingestion (100). It is metabolized within 15–29 h (101), but its continuous release and environmental persistence can disrupt the endocrine system, leading to reproductive disorders (102). *In vitro* studies showed TCS (0–10 μM) stimulated E2 and P secretion and upregulated steroidogenic genes in human granulosa KGN cells, indicating its endocrine-disrupting potential (103). Research by Wang et al. indicated that exposure to 0.16 mg/L TCS disrupts the hypothalamic–pituitary-ovarian axis in female Yellow River carp by increasing the synthesis and secretion of E2 and upregulating the hypothalamic mRNA expression levels (104).

### Air pollutants

The increasingly severe issue of air pollution, particularly its impact on health, has become a globally recognized concern, especially in developing countries (105, 106). Alongside national development, accelerated urbanization, expanded industrial production, and the prevalence of motorized transportation have significantly increased human exposure to harmful air pollutants (107). These pollutants, mainly including waste gases from industrial emissions (108), road dust, vehicle exhaust (109), and secondary pollutants (110) formed through complex reactions in the atmosphere such as PM (PM10 and PM2.5), Sulfur Oxides (SO_X_), and NO_X_, can penetrate the alveolar-capillary barrier, enter the bloodstream, and reach other tissues and organs (111).

Evidence from related mechanisms indicates that long-term inhalation of multiple air pollutants can lead to systemic inflammation (112), endothelial dysfunction (113), DNA methylation (114), and granulocyte apoptosis, thereby affecting the normal function of the reproductive system (114). The adverse health effects of PM may stem from its particulate characteristics (size, mass, and even shape) or the chemical components it adsorbs (115, 116). Its impacts on the human body primarily include cytotoxicity, oxidative stress, and inflammatory effects (117). By observing the cytotoxic effects of PM2.5 on Chinese hamster ovary cells over 24 h (118), it was found that PM can continuously activate the nuclear factor-kappa B signaling pathway, promoting the expression of apoptotic genes and proteins, and accelerating the apoptosis of ovarian granulosa cells and oocytes (119). Additionally, SO_2_, nitrogen dioxide (NO_2_), O_3_, and CO are all classified as “criteria” air pollutants by the U. S. Environmental Protection Agency (https://www.epa.gov/criteria-air-pollutants). People are often exposed to these pollutants simultaneously, making it difficult to determine the individual effects of specific pollutants. Current mechanistic research suggests that these pollutants may collectively exert negative impacts on the human reproductive system through various pathways such as hormonal disruption (120), oxidative stress induction, cellular DNA alterations (121), and epigenetic changes (122, 123).

### Heavy metal pollutants

Heavy metals, as a class of pollutants with persistent toxicity, originate from both human activities such as mining, coal burning, metal processing, and transportation, as well as natural phenomena like rock weathering and volcanic eruptions (124–126). In the environment, common heavy metal contaminants like Cd, Hg, Pb and chromium (Cr) can cause damage to human organs even at trace exposure levels (127). Heavy metals primarily enter the human body through contaminated drinking water, food intake (especially seafood) (128, 129), and smoking (130). Due to their resistance to biodegradation and their tendency to accumulate in organisms while being difficult to metabolize and excrete, heavy metals pose a long-term and continuous threat to human health (131).

Selenia Miglietta and her colleagues found that the levels of Pb and Cd within follicles might be associated with morphological changes in the ultrastructures (such as the endoplasmic reticulum, mitochondria, and nucleus) of oocytes and cumulus cells (132). These morphological changes could potentially lead to the arrest of oocyte maturation, thereby impairing their fertilization capabilities, weakening the steroidogenic activity of cumulus cells, and inducing cumulus cell apoptosis. Furthermore, exposure to multiple toxic metals [such as Cd, Hg, barium (Ba), and arsenic (As)] may also increase the risk of developing polycystic ovary syndrome (PCOS) by disrupting the endocrine system (133, 134).

Despite existing research revealing the association between environmental pollutants and ovarian function, there remain gaps in the depth and breadth of these studies. Current research often focuses on the effects of a single pollutant, lacking exploration of the synergistic effects of multiple pollutants and the cumulative effects of long-term exposure. Furthermore, there is a lack of systematic and comprehensive analysis regarding the effects of multiple pollutants on ovarian function. Therefore, this systematic review aims to address these research gaps by comprehensively evaluating the impact of overall and multiple environmental pollutants (including chemical, gaseous, and heavy metals) on female ovarian function. This review provides an in-depth assessment of pollutant-related reproductive toxicity, helping reduce the incidence of female infertility, support public health policies to protect women’s reproductive health, and providing reliable scientific evidence for clinical practice and policy-making.

## Materials and methods

### Search strategy and selection criteria

This systematic review was designed based on the Preferred Reporting Items for Systematic Review and Meta-Analysis Protocols (PRISMA-P) checklist (135) and the updated version of the Cochrane Handbook for Systematic Reviews (136). The review protocol was registered in the PROSPERO database (Registration number: CRD42024567744).

To comprehensively review the literature on the effects of environmental pollutants on ovarian function, we searched the following electronic databases: PubMed, EMBASE, Cochrane Library, and Web of Science. The search strategy is provided in Supplementary Table 1. The search covered all relevant studies from the inception of each database to July 30, 2024. Additionally, through this comprehensive search strategy, we aimed to expand the scope of the research and ensure the comprehensiveness and accuracy of the findings. During the literature screening process, the inclusion and exclusion criteria were as follows:

Inclusion Criteria: Women of reproductive age (menarche to premenopause, typically 15–49 years) (137) exposed to environmental pollutants (e.g., POPs, industrial waste, heavy metals); women with ovarian dysfunction, primary ovarian insufficiency, poor ovarian response, premature ovarian failure, or reduced ovarian reserve; studies reporting at least one primary outcome measure (e.g., antral follicle count [AFC], AMH, follicle-stimulating hormone [FSH], E2); cohort studies exploring the relationship between environmental pollutant exposure and ovarian function.

Exclusion Criteria: Women with ovarian dysfunction due to iatrogenic factors (including radiotherapy, chemotherapy, and medication), or lifestyle factors. Lifestyle-related ovarian dysfunction was determined based on predefined clinical criteria (138), including obesity [body mass index (BMI) ≥ 30] (139), excessive alcohol intake (>14 units/week) (140), or chronic sleep deprivation (141) with circadian dysregulation (142). Women of reproductive age with ovarian tumors, polycystic ovary syndrome, or other organic ovarian diseases; pregnant women; non-cohort studies, such as cross-sectional studies or case–control studies; studies not reporting relevant outcome measures; non-English publications (143); reviews, editorials, conference abstracts, and unpublished studies.

### Data extraction and quality appraisal

#### Data extraction

Three independent reviewers (LA, YH and YW) participated in the screening, eligibility assessment, and selection review process. Reviewer L. A. was responsible for downloading and conducting an initial screening to systematically review the literature and exclude studies irrelevant to the research question or not meeting the predefined criteria. Reviewers MZ, DW and SS and S. T. S. independently assessed the eligibility of the studies that passed the initial screening based on the predefined inclusion criteria. For studies with disagreements, reviewers YL and XL conducted further independent assessment and made the final decision to ensure scientific rigor and the validity of the selected studies.

After determining the eligibility of cohort studies, reviewers YH and YW independently completed data extraction. To ensure data consistency and accuracy, LA subsequently cross-checked a portion of the extracted data. All data were collected and organized using a pre-designed standardized Excel spreadsheet. Specific data extracted included: first author’s name, publication date, country of study, study design type, study objective, data source, sample size, type of exposure pollutants, mode of exposure measurement, characteristics of the study population (e.g., age, ethnicity), primary outcome measures, and relevant statistical indicators. All these details aimed to construct a comprehensive and accurate database to support further analysis of the impact of environmental pollutants on ovarian function.

#### Quality appraisal

Due to the significant differences in design and implementation between observational studies and randomized controlled trials, we used the NOS (144–146) and the Grading of Recommendations Assessment, Development and Evaluation (GRADE) (147–150) approach to systematically assess the quality and certainty of the evidence from cohort studies.

The NOS primarily evaluates studies in three domains: selection/exposure of study subjects, comparability between groups, and outcome measurement. High-quality studies were those with NOS scores of 8 or higher, medium-quality studies scored between 5 and 7, and low-quality studies scored below 5.

The three authors (LA, YH and YW) independently assessed the certainty of evidence for each type of compound’s impact on ovarian function using the GRADE framework. This assessment included evaluating the risk of bias at the outcome level, inconsistency, indirectness, imprecision, and other biases (such as publication bias or observational study design). GRADE evidence certainty levels: high (research unlikely to change confidence), moderate (research may importantly impact confidence), low (research very likely to impact confidence), and very low (estimates highly uncertain) (147). According to Murad and colleagues (151), the GRADE guidelines were appropriately applied to the narrative synthesis process. In doing so, we referred to Dr. Nafiso Ahmed’s team’s refined version of the GRADE scoring criteria (152), which was more carefully adapted and optimized to address the differences in research methods involved in each research question. The adaptations were discussed and agreed upon with the working group. For more detailed information, please consult Supplementary Table 2.

### Outcome criteria

The effects of environmental pollutants on ovarian function are complex and diverse (153). AMH, AFC, FSH, and E2 are important indicators of ovarian function and are highly sensitive to environmental pollutants, making them effective measures for assessing the potential damage of these pollutants on ovarian function (29, 154).

AMH is a hormone produced by small follicles in the ovary, mainly secreted by antral and pre-antral follicles, and serves as a stable indicator of ovarian reserve. A decrease in AMH levels indicates a decline in ovarian function (155–157). AFC refers to the number of antral follicles detected in the ovary via ultrasound, directly reflecting ovarian reserve. Therefore, higher AFC values indicate better ovarian reserve (158, 159). FSH is a hormone secreted by the pituitary gland that stimulates follicular development in the ovary and reflects the ovary’s response to stimulation. FSH is an important hormone for assessing ovarian function, and elevated FSH levels represent ovarian failure or decreased ovarian function due to negative feedback regulation (160). E2 is the primary estrogen secreted by the ovary, and its levels reflect follicular development and ovarian hormone secretion function, with abnormal changes indicating ovarian dysfunction (161, 162). Since AMH and AFC are relatively stable and highly correlated, the expression of AMH is barely affected by the cyclic fluctuations of gonadotropin, remaining relatively stable throughout the menstrual cycle (155, 163, 164). The most accepted predictor for female fertility is ovarian reserve (155). Therefore, in the GRADE framework, we evaluate them as direct evidence of ovarian function.

### Data analysis

Due to the complex and diverse effects of environmental pollutants, particularly the variation in chemical derivatives and study characteristics (e.g., study design, exposure dose, exposure duration, and participant demographics), conducting a meta-analysis presented certain challenges. Therefore, this study used a narrative synthesis approach, combining qualitative and quantitative data from existing literature to comprehensively understand the impact of these pollutants on ovarian function. The narrative analysis summarized the main findings of each study, analyze the sources of heterogeneity, explore the potential mechanisms of different environmental pollutants, and discussed the consistency and differences in study results. For studies with significant heterogeneity, specific factors influencing the outcomes were further examined to enhance the reliability of the analysis.

This study will focus on analyzing the specific effects of environmental pollutant exposure on ovarian function, summarizing and comparing reproductive indicators reported in different studies, including AMH, AFC, FSH, and E2, to evaluate differences or common trends in these indicators across studies. Quantitative analysis will also be conducted to assess the extent to which different types of environmental pollutants, such as BPA, PAEs, PCBs, and heavy metals (e.g., Pb, Cd), impact AMH, AFC, FSH, and E2.

## Results

**Figure 2:**
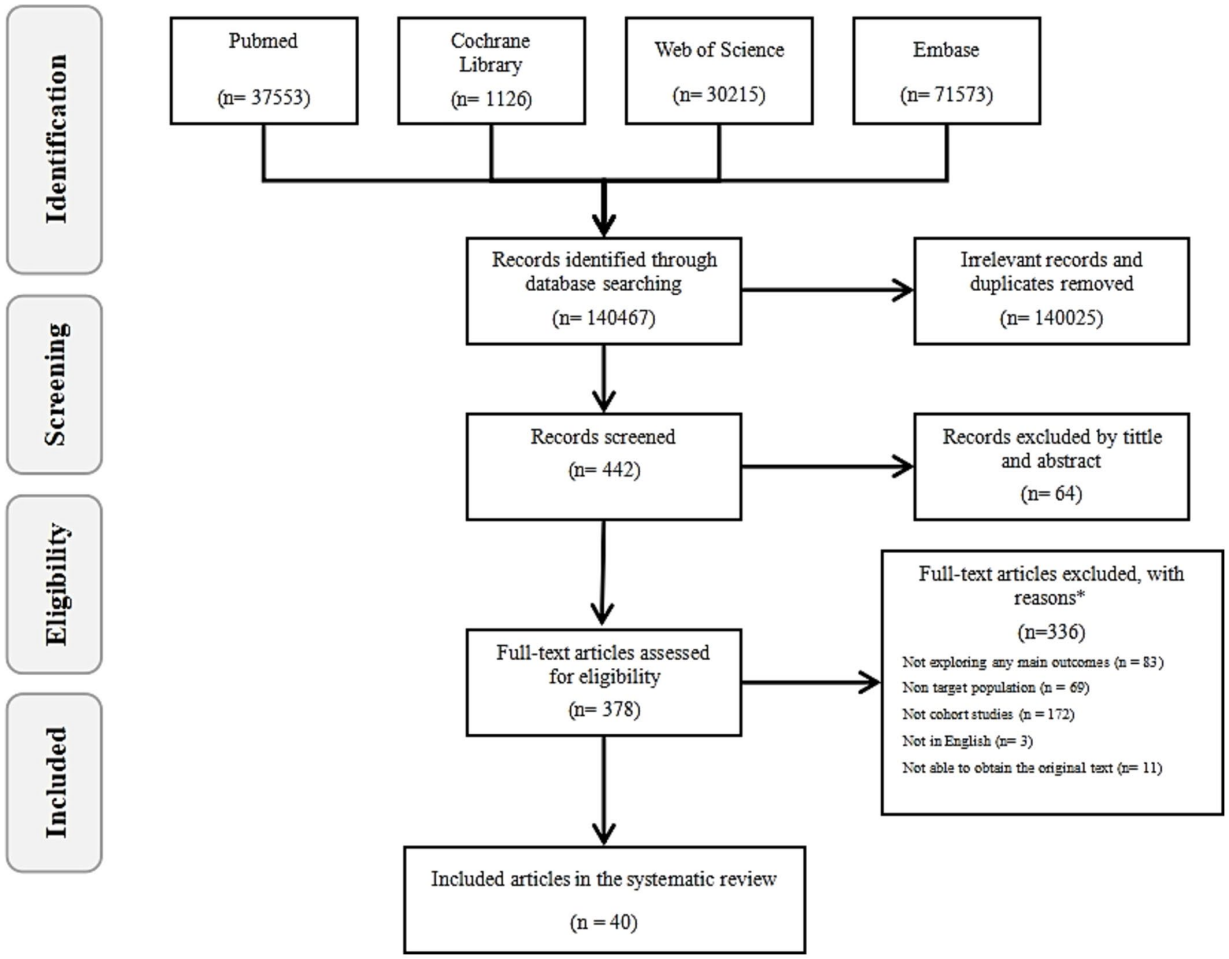
The flow diagram. By searching four databases (PubMed, Web of Science, Embase, Cochrane Library), the literature was screened according to the inclusion and exclusion criteria, and finally 40 records were included in the study.

A total of 140,467 results were initially obtained from four databases (PubMed, Web of Science, Embase, Cochrane Library). After removing duplicates and irrelevant entries (140,025), 442 articles remained for further screening (Figure 2). Of these, 64 were excluded at the title and abstract screening stage. Following a full-text review of remaining 378, 338 were excluded based on exclusion criteria, leaving 40 cohort study reports from 33 different studies that analyzed the effects of 20 types of pollutants on ovarian function. Of these 40 reports, 53% (21 studies) reported the effects of chemical industrial pollutants on ovarian reserve, including PFAS, PAEs, BPs, OCPs, PCBs, and other chemical pollutants. Additionally, 37% (15 studies) reported the effects of air pollutants on ovarian reserve, including gaseous pollutants (e.g., SO_X_, NO_X_, O_3_, Benzene, Toluene, Ethylbenzene, and Xylene [BTEX]) and PM (e.g., PM2.5, PM10). The remaining 10% (4 studies) reported the effects of heavy metals, including Hg, Cd, and Pb, on ovarian function.

### Description of studies

Supplementary Table 3 provides an overview of the characteristics of included studies. The 33 studies included women of reproductive age (18–54), with sample sizes ranging from 44 to 19,861 participants. These studies were conducted in the United States (*n* = 10), China (*n* = 8), Poland (*n* = 3), Italy (*n* = 2), South Korea (*n* = 2), Norway (*n* = 1), Denmark (*n* = 1), Sweden (*n* = 1), Israel (*n* = 1), India (*n* = 1), Iran (*n* = 1), Vietnam (*n* = 1), and South Africa (*n* = 1).

**Figure 3:**
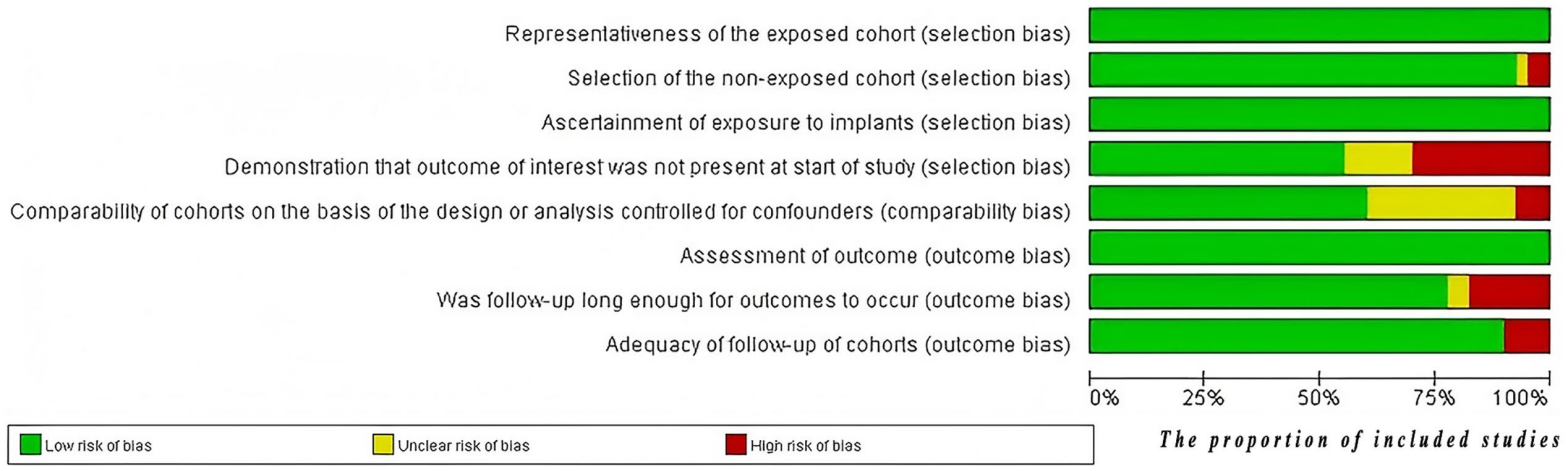
The risk of bias graph. The evaluations were conducted using the NOS, and the results figure was exported using RevMan software.

The quality of the studies was assessed using the NOS for cohort studies, with 27 studies (70%) rated as high quality and 13 studies (30%) rated as medium quality. Figure 3 presents the bias assessment results of these studies. The quality of evidence for each pollutant’s effect on ovarian function was independently assessed using the GRADE assessment method. Among the 20 pollutants studied, GRADE evidence quality was rated as high for 1 pollutant (5%), moderate for 3 pollutants (15%), and 16 pollutants (80%) as low to very low. Detailed information regarding the GRADE evidence profile, characteristics of the study population, NOS assessment of studies quality, and results of the subgroup analysis can be found in Supplementary Tables 2, 4–6.

### Association between chemical pollutants and ovarian function

The GRADE rating table is shown in Table 2.

**Table 2:** GRADE (grading of recommendations assessment, development and evaluation) evidence profile table.

No. of studies	Study quality	Inconsistency	Indirectness	Imprecision	Publication bias	Certainty
Polyfluoroalkyl Substances						
4	No concerns	Serious concerns	Borderline	Borderline	No concerns	Moderate certainty ⊕ ⊕ ⊕ ⊝
Phthalates						
7	No concerns	Borderline	No concerns	Serious concerns	No concerns	Very low certainty ⊕ ⊝⊝⊝
Benzophenones						
1	No concerns	No concerns	No concerns	Serious concerns	Serious concerns	Very low certainty ⊕ ⊝⊝⊝
Organochlorine Pesticides						
3	Serious concerns	Borderline	No concerns	Borderline	No concerns	Low certainty ⊕ ⊕⊝⊝
Polychlorinated Biphenyls						
2	Serious concerns	Serious concerns	No concerns	Serious concerns	No concerns	Very low certainty ⊕ ⊝⊝⊝
Bisphenol A						
3	Borderline	Borderline	Serious concerns	Serious concerns	No concerns	Very low certainty ⊕ ⊝⊝⊝
Parabens						
1	No concerns	No concerns	No concerns	Serious concerns	Serious concerns	Very low certainty ⊕ ⊝⊝⊝
Dioxin						
2	Borderline	Borderline	Serious concerns	Borderline	No concerns	Very low certainty ⊕ ⊝⊝⊝
Triclosan						
2	Borderline	Borderline	No concerns	Borderline	No concerns	Very low certainty ⊕ ⊝⊝⊝
PM2.5						
12	No concerns	Borderline	No concerns	No concerns	No concerns	High ⊕ ⊕ ⊕ ⊕
PM10						
9	No concerns	Serious concerns	No concerns	No concerns	No concerns	Low certainty ⊕ ⊕⊝⊝
SO_X_						
6	Borderline	Borderline	No concerns	No concerns	No concerns	Moderate certainty ⊕ ⊕ ⊕ ⊝
NO_X_						
10	Borderline	Serious concerns	No concerns	No concerns	No concerns	Low certainty ⊕ ⊕⊝⊝
O_3_						
7	Borderline	Serious concerns	No concerns	No concerns	No concerns	Very low certainty ⊕ ⊝⊝⊝
CO						
5	Borderline	Borderline	No concerns	No concerns	No concerns	Very low certainty ⊕ ⊝⊝⊝
BTEX						
1	No concerns	No concerns	No concerns	Serious concerns	Serious concerns	Very low certainty ⊕ ⊝⊝⊝
Tobacco smoke						
1	No concerns	No concerns	Serious concerns	Serious concerns	Serious concerns	Very low certainty ⊕ ⊝⊝⊝
Hg						
3	No concerns	Borderline	Borderline	No concerns	No concerns	Moderate certainty ⊕ ⊕ ⊕ ⊝
Cd						
1	No concerns	Borderline	Borderline	Serious concerns	Serious concerns	Very low certainty ⊕ ⊝⊝⊝
Pb						
1	No concerns	No concerns	Serious concerns	Serious concerns	Serious concerns	Very low certainty ⊕ ⊝⊝⊝

#### Polyfluoroalkyl substances

Four prospective studies explored the relationship between PFAS exposure and ovarian function, involving a total of 1,611 women. Of these studies, three were rated as “High” and one as “Medium” according to the NOS, with a GRADE certainty rating of moderate.

Björvang et al. (165) investigated a cohort of women undergoing *in vitro* fertilization (IVF) and found that the total PFAS levels in blood and follicular fluid (FF) were associated with higher baseline AFC, but a lower chance of obtaining high-quality embryos. The study also suggested that PFAS might be associated with lower AMH levels, although not significantly. This result was consistent with findings from Crawford et al. (166), who found no association between serum PFAS and AMH. Barrett et al. (167) discovered that PFAS exposure had different effects in nulliparous and parous women. Among nulliparous women, serum perfluorooctane sulfonate (PFOS) concentrations were significantly negatively correlated with salivary E2 (*β* = −0.025, 95% CI -0.043, −0.007), and these women showed higher sensitivity in ovarian hormone secretion to PFAS exposure compared to parous women. A longitudinal cohort study (168) also found that participants with higher serum PFAS concentrations had a shorter time to natural menopause, with FSH acting as a key mediator in the association between linear-chain perfluorooctanoate (n-PFOA), sum of branched-chain perfluorooctane sulfonate (Sm-PFOS) exposure, and natural menopause incidence, with total effect sizes of 26.9% (95% CI: 15.6, 38.4) and 13.2% (95% CI: 0.0, 24.5), respectively.

Summary: Although the specific strength of the associations varied across studies, the overall evidence consistently indicates that PFAS exposure may adversely affect ovarian function in women. Considering the prospective study designs, the moderate quality ratings from NOS and GRADE, and consistent findings across the studies, we can conclude that the weight of evidence is sufficient to support the conclusion that PFAS exposure poses a threat to female reproductive health.

#### Phthalates

Seven studies investigated the relationship between PAEs exposure and ovarian function in women, including six prospective studies and one retrospective study with a total of 2,334 participants. According to the NOS assessment, six studies were rated as “High” quality, and one as “Medium” quality, with GRADE evidence quality rated as low.

##### Evidence unrelated to ovarian parameters

The only retrospective study reported no association between PAEs and ovarian parameters. No significant dose–response relationship was observed between urinary PAEs metabolites and serum AMH levels (169).

##### Evidence of decreased ovarian parameters

Five prospective reports indicated that PAEs exposure was associated with a decline in ovarian parameters. Data from the Midlife Women’s Health Study (MWHS) (170) showed that higher cumulative exposure to urinary PAEs metabolites over the four-year study period was associated with lower E2 levels in year four. Mono-ethyl phthalate (MEP) and mono-benzyl phthalate (MBzP) contributed the most, with a doubling in MBzP concentration resulting in a 3.17% decrease in E2 levels (95% CI: −5.72, −0.55%). In the Environment and Reproductive Health (EARTH) study (171), a prospective association was observed between 11 urinary PAEs metabolites and AFC among women undergoing infertility treatment. Across the entire study cohort, higher quartiles of urinary DEHP metabolites were associated with lower AFC, particularly in women under 37 years.

An Indian study (172) found that women in the highest quartile of mono-iso-nonyl phthalate (MiNP) and mono-iso-decyl phthalate (MiDP) exposure had significantly lower AFC and serum AMH levels compared to those in the lowest quartile. Women in the highest quartile of mono-n-butyl phthalate (MBP) also exhibited a declining trend in E2 levels (*p* = 0.016). Higher levels of FF MEP were negatively correlated with average AFC and serum AMH levels, although these trends were not statistically significant. Beck et al. (173) reported that women in the highest tertiles of mono-iso-butyl phthalate (MiBP) and mono-n-butyl phthalate (MnBP) had an average of five fewer antral follicles compared to the reference group (*p* < 0.01).

Yao et al. (174) conducted an age-stratified analysis and found that, with similar levels of PAEs exposure, the relationship between PAEs metabolite concentration and AFC was positively correlated in women aged ≥35 years, whereas it was negatively correlated in women under 35 years. Among younger women, the second and third quartiles of MEP and ∑DEHP (sum of DEHP metabolites) were associated with a 6.50% (95% CI: −12.8, −0.18%) and 7.37% (95% CI: −13.8, −0.89%) reduction in AFC, respectively.

##### Evidence of increased ovarian parameters

Three studies mentioned that PAEs exposure could lead to increased ovarian parameters. A study from Denmark (173) found that women in the highest tertile of urinary mono-(2-ethyl-hexyl) phthalate (MEHP) had 26% higher E2 levels compared to those in the lowest tertile (95% CI: 5–51%, *p* = 0.02). Women in the highest tertiles of MiNP and MiDP showed significant increases in AMH levels by 44% (95% CI: 11–87%, *p* < 0.01) and an average increase of six antral follicles (95% CI: 2.14–9.00, *p* < 0.01), respectively. Hoffmann-Dishon et al. (175) observed a positive correlation between E2 levels in FF and MnBP/MiBP levels (*p* < 0.05). Additionally, Yao et al. (174) analyzed 525 women undergoing IVF treatment and found that MBP, mono(2-ethyl-5-oxohexyl) phthalate (MEOHP), and ∑PAEs (total PAEs) concentrations were positively correlated with AFC in women aged ≥35, whereas a negative correlation was observed in those younger than 35.

Summary: Across seven studies with varied geographic locations, time periods, and exposure ranges, the majority of high-quality prospective studies consistently demonstrated a decline in ovarian parameters, particularly in younger women (age < 35), suggesting a negative impact of PAEs on ovarian function. Although a definitive conclusion on the specific effects of PAEs on the ovaries has not yet been reached, existing evidence indicates that they have a clear disruptive effect on ovarian function. Given the widespread presence of PAEs in daily life and the potential risks of exposure, we should attach great importance to the potential health issues they may pose.

#### Benzophenones

Only one study examined the effect of BPs exposure on ovarian function in a cohort of 111 women. This study was rated as “High” quality by the NOS, with a GRADE evidence quality rating of very low.

This Danish study (173) investigated the association between the concentrations of six BPs in the 155 FF samples of 111 women undergoing IVF and fertility outcomes. It was found that women in the highest tertile of BP-3 had 30% higher E2 levels compared to the reference group (95% CI: 2%; 65%, *p* = 0.02). Notably, higher BP-3 levels were also significantly associated with a lower likelihood of live birth.

Summary: Currently, only one high-quality study from Denmark exists in this field. The positive correlation between BP-3 concentration and E2 levels observed in this study raises interest; however, this finding contrasts with the observed reduction in live birth rates among women with higher BP-3 levels, adding complexity to the interpretation. Despite the study’s high quality, the GRADE evidence quality rating was “low,” potentially reflecting limitations such as sample size. More high-quality, large-scale studies are needed to further verify and refine these findings.

#### Organochlorine pesticides

Three prospective studies investigated the effect of OCPs exposure on ovarian function, involving a total of 637 women. All three studies were rated as “Medium” quality by the NOS, with a GRADE evidence quality rating of low.

A Swedish study (165) analyzed nine OCPs in blood and FF and their association with assisted reproductive technologies (ART) outcomes. HCB in the blood was found to be significantly associated with lower AMH levels, negatively correlated with clinical pregnancy and live birth rates. However, the Study of Metals and Assisted Reproductive Technologies (SMART) (176), found that women with higher FF DDT concentrations had higher average E2 peak levels compared to women with lower DDT concentrations (1.45; 95% CI 0.92, 1.97; *p* < 0.0001). A study investigating the impact of serum OCPs on AMH levels in women from a South African village reported no evidence of an association between AMH levels and DDT exposure (177).

Summary: The three studies showed inconsistent results regarding the effect of OCP exposure on ovarian function. Due to the inconsistencies in the results, small sample sizes, regional limitations, and the overall low quality of evidence, no definitive conclusions can be drawn at this time.

#### Polychlorinated biphenyls

Two prospective studies investigated the effect of PCB exposure on ovarian function, involving a total of 217 women. Both studies were rated as “Medium” quality by the NOS, with a GRADE evidence quality rating of very low.

The SMART study (176) also analyzed the impact of PCBs on IVF outcomes in women, finding that women with high concentrations of PCB-151, PCB-170 and PCB-180 in FF had 47, 32 and 32% fewer baseline antral follicles, respectively, compared to women with low levels (*p* < 0.05). Additionally, women with high FF levels of PCB-138 and PCB-153 had E2 values 35 and 41% lower than those with low levels (*p* < 0.05). The study further revealed a significant association between PCB exposure and reduced embryo implantation rates. Conversely, Björvang et al. (165) found that PCBs exposure increased the number of antral follicles and enhanced ovarian responsiveness, but resulted in a decline in embryo quality.

Summary: The evidence suggests that PCBs negatively impact ovarian function, but the evidence quality from existing prospective studies is very low. Although high concentrations of PCB-151, PCB-170, and PCB-180 were associated with fewer antral follicles, methodological limitations and small sample sizes necessitate cautious interpretation of these findings.

#### Bisphenol A

Three prospective studies investigated the effect of BPA exposure on ovarian function, involving a total of 274 women. Of these, two were rated as “High” quality and one as “Medium” by the NOS, with a GRADE evidence quality rating of very low.

Mok-Lin et al. (178) found a positive correlation between serum E2 levels and total oocyte count (*p* < 0.001). However, each log unit increase in specific gravity-BPA was associated with a 12% decrease in oocyte count (*p* = 0.007) and a 213 pg./mL reduction in E2 peak (*p* = 0.03). These findings are consistent with the SMART study (179), which found a negative correlation between BPA and peak E2 levels (*p* = 0.06), although BPA was not associated with AFC or FSH levels. In contrast, a study from South Korea (180) did not find any significant effect of BPA on E2 levels or embryo quality.

Summary: Mok-Lin et al. and the SMART study observed negative associations between BPA levels and both oocyte count and E2 peak levels, although these findings were not consistent across all markers, as BPA was not linked to AFC or FSH levels. Additionally, the South Korean study found no impact of BPA on E2 levels or embryo quality. Therefore, while some evidence suggests that BPA may negatively impact ovarian function, the overall evidence is not entirely consistent and may be influenced by various factors. Comprehensive analysis indicates that BPA may have adverse effects on ovarian function; however, the quality of current evidence is low. This conclusion is primarily constrained by the overall insufficient sample size in the studies, which subsequently leads to a lower rating in the GRADE evidence quality evaluation system.

#### Parabens

Only one study reported on the impact of parabens exposure on ovarian function in a cohort of 192 women, which was rated as “High” quality by the NOS, with a GRADE evidence quality rating of very low.

A report from the EARTH study (181) analyzed the association between urinary parabens concentrations and ovarian reserve markers. Methylparaben (MP) and propylparaben (PP) were detected in over 99% of urine samples. Researchers observed a decreasing trend in AFC in women with higher PP concentrations (*p* = 0.07), and an increasing trend in day-3 FSH levels, consistent with the negative correlation between FSH and AFC (*p* = 0.002).

Summary: This study indicated that high urinary PP concentrations were associated with a decreasing trend in AFC and an increasing trend in FSH levels, although the association did not reach conventional significance levels. This finding suggests the potential adverse impact of PP on ovarian reserve function.

#### Dioxins

Two studies analyzed the effect of dioxins exposure on ovarian function in women, one prospective and one retrospective with a total of 472 participants. The prospective study was rated as “High”quality and the retrospective study as “Medium” by the NOS, with both studies receiving a GRADE evidence quality rating of very low.

One study analyzed the relationship between ovarian function and serum levels of TCDD in women who had been exposed to high concentrations of TCDD 20 years earlier (182). The other study investigated the impact of TCDD by analyzing steroid hormone levels in saliva and serum from women in high-exposure and low-exposure regions (183). However, neither study found conclusive evidence of a significant effect of TCDD on ovarian function.

Summary: The first study suggested a potential impact of TCDD on female reproductive health, while the second study analyzed the relationship between steroid hormone levels and TCDD exposure, but could not conclusively demonstrate a direct effect on ovarian function. Although no definitive conclusions were drawn, these studies provide important clues for understanding dioxins’ potential risks to the human endocrine system and reproductive health.

#### Triclosan

Two prospective studies investigated the effect of TCS exposure on ovarian function, involving a total of 620 women. One study was rated as “High” quality by the NOS, and the other as “Medium,” with a GRADE evidence quality rating of very low.

Jurewicz et al. (184) explored that urinary TCS concentrations significantly reduced AFC (*p* = 0.03), but no associations were observed between TCS exposure and other parameters such as E2, FSH, and AMH levels. Additionally, the EARTH study (185) found a negative correlation between specific gravity-adjusted urinary TCS concentration and AFC (*p* = 0.04) with younger (<35 years) and leaner (BMI < 25 kg/m^2^) women experienced a greater decline in AFC.

Summary: Both prospective studies indicated that TCS exposure may have adverse effects on ovarian function, as shown by the negatively correlation between urinary TCS concentrations and ovarian function parameters. These findings provide valuable insights into this area of research.

### Air pollutants and ovarian function

#### Particulate matter

Fourteen articles analyzed the impact of PM (PM2.5 and PM10) exposure on ovarian function in women, derived from thirteen different studies. These studies included 54,348 participants for PM2.5 analyses and 45,410 participants for PM10 evaluations. Ten of the studies were rated as “High” quality by the NOS, while three were rated as “Medium.” The GRADE evidence quality for PM2.5 was rated as high, whereas for PM10 it was rated as low.

Seven studies (186–193) consistently found a significant association between PM2.5 or PM10 exposure and decreased AFC and AMH levels. The negative impact of PM2.5 on ovarian reserve was equivalent to aging by approximately two years (186). In women diagnosed with infertility, the impact of PM10 on AFC was particularly pronounced (188). Research teams led by Wang (192) and Pang (190) highlighted that the impact of PM2.5 on AMH was especially prominent during the early transition from primary follicles to preantral follicles. However, there were divergent findings regarding the age-dependent impact of PM2.5: La Marca et al. (193) observed that the negative effect of PM2.5 on AMH diminished with age; Xinyan Wang et al. (192) demonstrated that stronger inverse associations were observed in women <35 years; Lanlan Fang et al. found that after age stratification, PM2.5/PM10 exposure had significantly stronger negative effects on E2 and FSH levels in older women (>30 years) (*p* < 0.05) (194); whereas Wieczorek (188) study suggested that older women (>35 years) were more affected by PM2.5, with more pronounced declines in AFC and AMH.

One study found a monotonic decrease in FSH levels with PM2.5 and PM10 exposure, along with a “U”-shaped exposure-response curve for E2 levels (194). Another study reported that women with higher PM10 exposure levels in the early luteal phase had higher E2 levels (*p* = 0.02) (195). Nevertheless, four studies (196–199) found no significant association between PM2.5 or PM10 exposure and ovarian reserve markers. For example, LaPointe et al. (197) did not observe any correlation between PM2.5 and AFC, but noted that high PM2.5 exposure led to reduced total oocyte counts, mature/metaphase II oocyte numbers, and ovarian sensitivity index.

Summary: PM2.5 exposure appeared to have a broader and more profound impact on ovarian reserve function, affecting not only AMH and AFC but also oocyte quantity and quality, while PM10 effects seemed to be more specific, notably impacting women with infertility and affecting E2 levels.

#### Gaseous pollutants

SO_X_, NO_X_, O_3_, carbon monoxide (CO), BTEX, and tobacco smoke are common gaseous pollutants, with SO_X_ and NO_X_ most commonly appearing in the form of SO_2_ and NO_2_.

Thirteen studies analyzed the effect of gaseous pollutants exposure on ovarian function in women, each contributing one article. The analyses included 29,243 participants for SOx, 50,939 for NOx, 48,882 for O3, 23,720 for CO, 806 for BTEX, and 132 for tobacco smoke exposure. The NOS quality ratings showed that eight studies were rated as “High” and five as “Medium.” The GRADE quality ratings and specific outcomes for the impact of various gaseous pollutants on ovarian function are as follows:

Moderate-quality evidence (188, 191, 194, 199) suggests that SO_X_ has a predominantly negative correlation with ovarian markers such as AFC, AMH, and FSH, with stronger negative associations observed in older women (>30 years) (194), although some studies (189, 198) did not find a significant association between SO_2_ and AMH. Very low-quality evidence (188–191, 193, 196–199) shows that NO_X_ have little effect on AMH and AFC, with only one study (194) indicating a positive correlation between NO_2_ and E2, FSH. Very low-quality evidence (189, 190, 194, 199) indicates that O_3_ generally shows no association with ovarian reserve markers, although a few studies reported a negative correlation between O_3_ and AMH (192, 200), and a positive correlation with FSH and E2 (194). Very low quality evidence (189, 197, 199) did not find a significant association between CO and ovarian reserve, except for one study suggesting a negative correlation between CO and FSH, E2 (194). Very low-quality evidence (198) for BTEX suggests no significant association with AMH levels, while very low quality evidence (195) for tobacco smoke indicates a significant association with increased E2 levels.

Summary: Findings reveal considerable variability in the impact of different gaseous pollutants on ovarian function in women. SO_X_ generally shows a negative correlation with ovarian function markers, while the effects of NO_X_, O_3_, and CO are inconclusive. Tobacco smoke may be associated with increased E2 levels.

### Heavy metals and ovarian function

Due to the limited availability of cohort studies on the impact of heavy metals on ovarian function, this section provides an overview without detailed subdivision. Three independent studies (reported in four articles) were identified examining Cd, Hg, and Pb exposure. These analyses included 810 participants for Hg exposure, 525 for Cd exposure, and 525 for Pb exposure. The NOS quality ratings showed that two studies were rated as “High” and one as “Medium.” The GRADE quality ratings and specific outcomes for their impact on ovarian function are as follows:

Moderate-quality evidence suggests mixed findings regarding the impact of Hg on ovarian reserve, with one out of three studies showed a positive correlation between Hg and AFC (201), while the other two did not find a significant association (202, 203). Very low-quality evidence (203, 204) suggested a positive correlation between Cd and AMH and E2 levels. Another study with very low-quality evidence (203) found no significant correlation between Pb and ovarian markers. The study by Mínguez-Alarcón et al. (201) a positive correlation was observed between hair Hg and AFC, specifically among women consumed more than 0.125% of their total caloric intake from n-3 polyunsaturated fatty acids (n3PUFA) weekly. In this study higher Hg tertiles were associated with an increasing trend in AFC (*p* = 0.004), it is possible that the higher exposure group consumed more fish, which may reflect the positive effect of n3PUFA in fish rather than the impact of the heavy metal itself (201–203). In addition, the research by Kim (204) found that higher blood Cd concentrations led to increased testosterone and AMH levels, with each 0.1 μg/L rise in blood Cd increasing the probability of a mild polycystic ovarian syndrome (PCOS) phenotype by 18% (RR 1.18; 95% CI 1.06, 1.31).

Summary: The impact of heavy metals on ovarian function is a complex and relatively understudied area. Existing evidence on the direct association between heavy metals and ovarian function remains contentious and may be influenced by other factors, such as n3PUFA intake, making it difficult to clearly delineate the effect of heavy metals on ovarian function. Therefore, more high-quality studies are needed to explore this area in depth.

## Discussion

This review synthesizes findings from 33 cohort studies, covering twenty different categories of environmental pollutants. These pollutants, widely present in various consumer products, were explored their associations with ovarian function, with GRADE assessments indicating that PFAS, PAEs, TCS, PM, and SO_X_ have the clearest evidence of impact on ovarian reserve. Specific manifestations include: PFAS significantly reduces AMH levels and suppresses E2 secretion; PAEs correlate with diminished AMH and AFC; elevated TCS concentrations are linked to reduced AFC; PM2.5/PM10 exposure leads to declines in AFC and AMH; while SOX broadly disrupts ovarian biomarkers (AFC, AMH, FSH, Supplementary Table 7 summarizes and correlates the effects of these pollutants on ovarian function with their potential mechanisms of action). However, due to the limited number of studies and the heterogeneity in study designs, the impact of other pollutants remains inconclusive.

Chemical pollutants are particularly relevant due to their diverse forms and frequent contact in daily life, especially for women, who may be more exposed to personal care products than men. The negative impact of these chemical pollutants on the ovaries is substantial. They not only disrupt normal ovarian hormone secretion but also impede proper oocyte development. Multiple studies have indicated that various pollutants like PAEs (such as DEHP, MiBP, and MnBP) (171, 173), PCBs (specifically PCB-151, PCB-170, PCB-180) (176), parabens (PP) (181), and TCS (184, 185) adversely affect ovarian reserve ovarian reserve by inhibiting antral follicle growth, causing follicular cell cycle arrest and increasing follicle atresia. This impact is especially pronounced in younger women (<35 years) (185, 188), aligning with findings (181) that elevated urinary PP levels are associated with increased day-3 FSH levels and reduced AFC, which closely resembles clinical features of premature ovarian insufficiency (205). Although some PAEs metabolites (such as MEOHP and ∑PAEs) (174), PFAS (165), and certain PCBs (PCB-118) (165) may increase AFC. This likely reflects accelerated recruitment of primordial follicles, potentially promoting early follicle depletion. Animal studies (174, 206) support this view as well: exposure to di-n-butyl phthalate (DBP), the precursor of MBP, promotes the depletion of follicular follicles by accelerating primordial follicle recruitment in rats. These observations suggest that a higher baseline AFC in exposed individuals does not correlate with improved fertility outcomes, as pollutants also lower embryo quality during IVF (165), revealing the complex and far-reaching impact of these pollutants on ovarian health. Moreover, these pollutants exhibit estrogenic effects, can bind to estrogen receptors (ER) alpha and ER beta, thereby influencing the expression of endogenous estrogen to some extent (207).

Among air pollutants, PM2.5 is the most clearly harmful to ovarian function, followed by PM10, both of which directly affect ovarian reserve. Due to it is smaller in diameter, PM2.5 is more easily to penetrate the alveolar-capillary barrier and reaches the ovaries. The organic matter, soluble metals, and other substances contained within PM2.5 can interfere with ovarian function (208, 209), with polycyclic aromatic hydrocarbons (PAHs) being particularly representative (195). PAHs exposure can lead to accelerated loss of primordial follicles, resulting in premature ovarian reserve depletion (210). The effects of PAHs exposure may also extend to subsequent generations, leading to reduced ovarian reserve.

Research on heavy metals and ovarian function is limited, but numerous animal studies have emphasized the reproductive toxicity of heavy metals (211) like Cd, Hg and Pb, which do not metabolize and are toxic at any concentration (212). Humans are primarily exposed through contaminated water, seafood, and smoking (212). However, individuals with high exposure tend to consume more fish (201–203), and the positive effects of n3PUFA in fish on ovarian reserve may mask the adverse effects of heavy metals. Therefore, future research needs to employ more rigorous experimental designs to investigate the impact of heavy metals on ovarian function.

### Limitations and strengths of the study

#### Study design limitations

We systematically integrated comprehensive reports from 33 cohort studies, most of which (*N* = 20) that remained after screening were infertile patients, potentially limiting generalizability to the broader population. On the other hand, this highlights the potential correlation between environmental pollutants and infertility, warranting further research (213, 214). Additionally, the observed contradictions in the association between environmental pollutant exposure and ovarian function across studies may be attributed to several factors. First, the magnitude of exposure (low vs. high) and duration of exposure may influence outcomes, as populations exposed to high levels of environmental pollutants (e.g., those living near industrial areas) may experience different health effects than those with background exposure (215, 216). Second, methodological variations among studies may lead to inconsistent results. These include differences in study design (e.g., cross-sectional vs. longitudinal), sample size, methods for measuring environmental pollutants (e.g., blood serum, follicular fluid, urine), and statistical approaches (e.g., adjusting for different confounders). Future studies would benefit from standardized exposure assessment protocols, such as harmonized biomonitoring matrices (e.g., urine vs. serum) and consistent quantification methods. Moreover, the timing of sample collection (e.g., follicular phase vs. luteal phase of the menstrual cycle) can significantly impact hormone measurements (195), potentially masking or exaggerating associations (217, 218). Third, exposure variations may contribute to these inconsistencies. Different types of environmental pollutants have distinct chemical properties, metabolic pathways, and biological effects, which may result in varying impacts on ovarian function. Furthermore, differences in study populations could play a critical role. The age, BMI, lifestyle factors (e.g., smoking, alcohol consumption), and reproductive history (nulliparous vs. parous) may modify the effects of environmental pollutants on ovarian health. For example, age differences further influenced outcomes (200, 219), as ovarian decline in women aged ≥35 years may obscure pollutant effects, particularly with chemical pollutants and PM (190, 191, 220). Notably, for certain compounds (e.g., BPs and PCBs), the limited number of related studies resulted in a weak evidence base, making it challenging to comprehensively assess their effects on ovarian function. To address these limitations, we recommend prioritizing longitudinal cohort studies with repeated exposure measurements and multi-country collaborative designs to account for geographic variability in pollutant profiles. By recognizing and analyzing these factors, we can better understand the complexity of the relationship between environmental pollutants and ovarian function and identify areas for future research.

#### Strengths of the study

Compared with previous research, this review has several significant strengths. Firstly, it included pollutants from various exposure pathways such as air, water, and soil, enhancing the overall understanding of the impact of environmental pollutants on ovarian function. Secondly, this study covered all relevant articles from the establishment of the database to the present day, and multiple geographical regions, reflecting differences in exposure across locations. The multidimensional analysis of ovarian function evaluation indicators (e.g., AMH, AFC, FSH, E2) further enhanced understanding of the impact of pollutants on ovarian reserve and function. Lastly, this review systematically assessed the quality of the included studies used the NOS and the GRADE framework, increasing the robustness and reliability of the conclusions.

### Policy and practice recommendations

Based on the study findings, it is recommended that public health regulations be strengthened concerning certain chemical pollutants (e.g., PCBs, PFAS), particularly to protect pregnant and reproductive-age women (221–223). Existing frameworks such as the EU REACH regulations (224, 225) and US EPA PFAS Action Plan (226) have demonstrated effectiveness in reducing exposure to legacy pollutants through restrictions on production and environmental discharge. However, we identifies emerging contaminants (e.g., BPA) that currently fall outside these regulatory frameworks despite evidence of ovarian toxicity (227). Policies should prioritize not only reducing exposure to individual pollutants but also investigating the combined and cumulative effects of multiple contaminants (228), as mandated by the 2021 EU Chemicals Strategy for Sustainability (229). Public education initiatives must be strengthened to encourage lifestyle and dietary habits that minimize pollutant exposure, modeled after successful programs like the US National Biomonitoring Program’s community outreach (230). Enhancing dynamic biomarker monitoring can further improve the accuracy of long-term exposure assessments.

Clinically, these findings emphasize the need for proactive measures to protect reproductive-age women from environmental pollutants. Regular ovarian health assessments, tailored guidance, and advanced technologies such as AI-driven exposure tools (231) are crucial for identifying high-risk individuals and implementing targeted prevention strategies. Practical steps, including avoiding plastic products, reducing air pollution exposure, and selecting uncontaminated food, can significantly lower risks (232). Physicians should educate patients, particularly women in reproductive age, planning pregnancy or women at higher risk due to occupational and environmental factors, on minimizing pollutant exposure and leveraging early screening methods for ovarian dysfunction (233).

For researchers, future research should expand sample sizes to include women of different ages and reproductive states to clarify the long-term impact of these pollutants on ovarian reserve, incorporating longer follow-up periods and more precise exposure assessments. More attention should also be given to research on male exposure to gain a comprehensive understanding of the impact of environmental pollutants on couple fertility (234). Rigorous study designs, especially for heavy metals, should control for confounders like fish consumption. This review also highlights gaps in the literature regarding the effects of some pollutants on ovarian function, including the long-term effects of pollutants with long half-lives and the mechanisms of pollutant impact on ovarian function, which urgently need more high-quality research to provide policy-relevant evidence, particularly for regulatory agencies evaluating chemical prioritization under programs like the US EPA’s Toxic Substances Control Act (235).

## Conclusion

This systematic review highlights the substantial impact of environmental pollutants on ovarian function, emphasizing the role of chemical pollutants (e.g., PFAS, PAEs, PCBs), air pollutants (e.g., PM2.5, PM10), and heavy metals (e.g., Pb, Cd) in reducing ovarian reserve and impairing hormonal function. Women of reproductive age, particularly those exposed to high levels of these pollutants, face increased risks of infertility.

The review reveals significant gaps in current research, particularly in understanding the cumulative and synergistic effects of multiple pollutants and their long-term health outcomes. These findings advocate for stricter public health policies, enhanced education on minimizing exposure, and advanced monitoring technologies to protect reproductive health. For clinical practice, regular ovarian health assessments and personalized advice on reducing pollutant exposure are essential to mitigate risks. Future research should prioritize rigorous, large-scale studies exploring the molecular mechanisms of pollutant-induced ovarian dysfunction to develop effective preventive and therapeutic strategies.

## Data Availability

The original contributions presented in the study are included in the article/Supplementary material, further inquiries can be directed to the corresponding authors.
