# Supplementary material for: Assessment of ovarian dysfunction induced by environmental toxins: a systematic review
Source: Front Public Health. 2025 Jul 30;13:1575418. doi: 10.3389/fpubh.2025.1575418 (PMC12343636; doi:10.3389/fpubh.2025.1575418)
Supplement: Supplementary file 1 [file Table_1.docx]

Supplementary Table A.1 The table of search strategy

| #1 | "Microplastics"[Mesh] |
| --- | --- |
| #2 | "Polystyrenes"[Mesh] |
| #3 | "Polypropylenes"[Mesh] |
| #4 | "Polyethylene"[Mesh] |
| #5 | "Metals, Heavy"[Mesh] |
| #6 | "Mercury"[Mesh] |
| #7 | "Chromium"[Mesh] |
| #8 | "Nickel"[Mesh] |
| #9 | "Copper"[Mesh] |
| #10 | “Lead"[Mesh] |
| #11 | "Persistent Organic Pollutants"[Mesh] |
| #12 | Pollutant, Persistent Organic[Title/Abstract] |
| #13 | “Industrial Waste”[MeSH] |
| #14 | "Soil Pollutants"[Mesh] |
| #15 | Pollutant, Soil[Title/Abstract] |
| #16 | "Water Pollutants"[Mesh] |
| #17 | Pollutant, Water[Title/Abstract] |
| #18 | "Endocrine Disruptors"[Mesh] |
| #19 | Disruptor, Endocrine[Title/Abstract] |
| #20 | Chemical, Endocrine Disrupting[Title/Abstract] |
| #21 | Endocrine Disruptor Effect[Title/Abstract] |
| #22 | Disruptor Effect, Endocrine[Title/Abstract] |
| #23 | "Polychlorinated Dibenzodioxins"[Mesh] |
| #24 | "DDT"[Mesh] |
| #25 | "Fluorocarbons"[Mesh] |
| #26 | "Polychlorinated Biphenyls"[Mesh] |
| #27 | "Phthalic Acids"[Mesh] |
| #28 | "Triclosan"[Mesh] |
| #29 | "Genistein"[Mesh] |
| #30 | "Benzene Derivatives"[Mesh] |
| #31 | "Air Pollution"[Mesh] |
| #32 | "Air Pollutants"[Mesh] |
| #33 | Pollutant, Air[Title/Abstract] |
| #34 | Air Pollutants, Environmental[Title/Abstract] |
| #35 | Environmental Pollutants, Air[Title/Abstract] |
| #36 | Pollutants, Air Environmental[Title/Abstract] |
| #37 | PM2.5[Title/Abstract] |
| #38 | PM10[Title/Abstract] |
| #39 | air quality[Title/Abstract] |
| #40 | #1 or #2 or #3 or #4 or #5 or #6 or #7 or #8 or #9 or #10 or #11 or #12 or #13 or #14 or #15 or #16 or #17 or #18 or #19 or #20 or #21 or #22 or #23 or #24 or #25 or #26 or #27 or #28 or #29 or #30 or #31 or #32 or #33 or #34 or #35 or #36 or #37 or #38 or #39 |
| #41 | "Ovarian Reserve"[Mesh] |
| #42 | ovarian function[Title/Abstract] |
| #43 | antral follicle count[Title/Abstract] |
| #44 | "Anti-Mullerian Hormone"[Mesh] |
| #45 | "Gonadal Hormones"[Mesh] |
| #46 | "Ovary"[Mesh] |
| #47 | "Ovarian Follicle"[Mesh] |
| #48 | "Gonads"[Mesh] |
| #49 | "Corpus Luteum"[Mesh] |
| #50 | "Primary Ovarian Insufficiency"[Mesh] |
| #51 | Ovaries[Title/Abstract] |
| #52 | Follicle, Ovarian[Title/Abstract] |
| #53 | Ovarian Follicles[Title/Abstract] |
| #54 | Follicle, Graafian[Title/Abstract] |
| #55 | Follicle, Atretic[Title/Abstract] |
| #56 | "Follicular Fluid"[Mesh] |
| #57 | Fluid, Follicular[Title/Abstract] |
| #58 | Folliculi, Liquor[Title/Abstract] |
| #59 | Antral Fluid, Ovarian Follicle[Title/Abstract] |
| #60 | Gonad[Title/Abstract] |
| #61 | Corpora Lutea[Title/Abstract] |
| #62 | Premature Ovarian Failure[Title/Abstract] |
| #63 | Gonadotropin Resistant Ovary Syndrome[Title/Abstract] |
| #64 | Fragile X Associated Primary Ovarian Insufficiency[Title/Abstract] |
| #65 | Resistant Ovary Syndrome[Title/Abstract] |
| #66 | Primary Ovarian Insufficiency, Fragile X Associated[Title/Abstract] |
| #67 | Fragile X Premature Ovarian Failure[Title/Abstract] |
| #68 | Premature Ovarian Failure 1[Title/Abstract] |
| #69 | FMR1 Related Primary Ovarian Insufficiency[Title/Abstract] |
| #70 | Hypergonadotropic Ovarian Failure, X Linked[Title/Abstract] |
| #71 | Premature Ovarian Failure, X Linked[Title/Abstract] |
| #71 | X Linked Hypergonadotropic Ovarian Failure[Title/Abstract] |
| #72 | "Disorders of Sex Development"[Mesh] |
| #73 | Ovarian dysfunction[Title/Abstract] |
| #74 | diminished ovarian reserve[Title/Abstract] |
| #75 | poor ovary response[Title/Abstract] |
| #76 | premature ovarian failure[Title/Abstract] |
| #77 | #41 or #42 or #43 or #44 or #45 or #45 or #46 or #47 or #48 or #49 or #50 or #51 or #52 or #53 or #54 or #55 or #56 or #57 or #58 or #59 or #60 or #61 or #62 or #63 or #64 or #65 or #66 or #67 or #68 or #69 or #70 or #71 or #72 or #73 or #74 or #75 or #76 |
| #78 | #40 and #77 |
