# Supplementary material for: Assessment of ovarian dysfunction induced by environmental toxins: a systematic review
Source: Front Public Health. 2025 Jul 30;13:1575418. doi: 10.3389/fpubh.2025.1575418 (PMC12343636; doi:10.3389/fpubh.2025.1575418)
Supplement: Supplementary file 3 [file Table_3.docx]

Supplementary Table A3. The Table of Study Characteristics for Included Studies

| **First author (year)** | **Study Name /Acronym** | **Country/ Region** | **Study Design** | **Specific population, condition, domain or measured symptom being studied** | **No. Women** | **Sample/ Mode of exposure measurement** | **Ovarian outcome/ Tested samples** | **NOS/ Quality assessments** |
| --- | --- | --- | --- | --- | --- | --- | --- | --- |
| (Barrett et al., 2015) | The EBBA-I Study | Tromsø, Norway | Prospective Cohort Study | General Population: only women | Baseline: 207  Follow up: 178 | PFASS: serum | E2, P: saliva | 8/ high |
| (Beck et al., 2024) | Unnamed Survey | Denmark | Prospective Cohort Study | Infertile couples undergoing IVF | 111 | Phthalates: follicular fluid | Reproductive hormones: follicular fluid AFC: transvaginal ultrasonography | 8/ high |
| (Björvang et al., 2022) | Unnamed Survey | Uppsala, Sweden | Prospective Cohort Study | Women undergoing assisted reproductive technology | 185 | OCPs, PCBs, PBDEs, and PFASs: serum, follicular fluid | AMH: serum AFC: ultrasound | 7/ medium |
| (Bloom et al., 2011) | The SMART Study | San Francisco, USA | Prospective Cohort Study | Women undergoing IVF | 44 | BPA: serum | E2: serum The number of oocytes retrieved: transvaginal ultrasonography | 7/ medium |
| (Bloom et al., 2017) |  | San Francisco, USA | Prospective Cohort Study | Female patients undergoing a 1st completed IVF cycle | 32 | PCB, DDT, DDE: follicular fluid | Reproductive hormone: serum AFC, follicular response: transvaginal ultrasound Clinical IVF outcomes: serum hcg test | 6/ medium |
| (Crawford et al., 2017) | Unnamed Survey | Raleigh, Durham, and Chapel Hill, North Carolina area, USA | Prospective Cohort Study | Women without infertility | 99 | PFCS: serum | AMH: serum | 8/ high |
| (Ding et al., 2022) | The SWAN Study | USA | Longitudinal Cohort Study | Premenopausal women | 1,120 | PFAS: serum | FSH, E2: serum incidence of natural Menopause: self-report | 8/ high |
| (Fang et al., 2023) | Unnamed Survey | Anhui, China | Longitudinal Cohort Study | Female partners from the longitudinal assisted reproduction cohort in Anhui, China | 19861 | PM2.5, PM10, SO_2_, CO, NO_2_, O_3_: collected from China High Air Pollutants | E2, P, T, FSH: serum | 7/ medium |
| (Gaskins et al., 2019) | The EARTH Study | USA | Prospective Cohort Study | 632 women undergoing infertility treatment | 632 | PM2.5: estimated with a validated hybrid model of satellite-derived aerosol optical depth measurements and land-use terms | AFC: transvaginal ultrasonography | 8/ high |
| (Hood et al., 2021) |  | USA | Prospective Cohort Study | Women undergoing infertility evaluation and treatment | 565 | PM2.5: used a validated hybrid model of satellite and land use data with a 1 km2 spatial resolution | AFC: transvaginal ultrasonography | 8/ high |
| (Smith et al., 2013) |  | Boston, Massachusetts, USA | Prospective Cohort Study | Women undergoing fertility treatment | 192 | Parabens: urine | AFC, Ovarian volume: transvaginal ultrasound FSH: serum | 8/ high |
| (Mínguez-Alarcón et al., 2021) |  | USA | Prospective Cohort Study | Women attending a fertility center | 353 | Hg: hair | AFC: transvaginal ultrasonography | 9/ high |
| (Mínguez-Alarcón et al., 2017) |  | USA | Prospective Cohort Study | Women seeking care at a fertility center | 109 | TCS: urine | AFC: transvaginal ultrasonography | 9/ high |
| (Messerlian et al., 2016) |  | USA | Prospective Cohort Study | Women seeking infertility investigation and treatment | 215 | Phthalate metabolites: urine | AFC: transvaginal ultrasonography | 9/ high |
| (Feng et al., 2021) | Unnamed Survey | Shanxi, China | Retrospective Cohort Study | 600 women in Reproductive Medical Center | 600 | SO_2_, NO_2_, PM10, PM2.5, CO, O_3_: estimated based on the air pollutants monitoring data in the eleven prefectural-level cities | AFC: transvaginal ultrasonography | 7/ medium |
| (Du et al., 2018) | Unnamed Survey | Wuhan, China | Retrospective Cohort Study. | Women aged between 20 and 45 years, with indications for IVF or ICSI | 415 | Phthalate metabolites: urine | AMH, INHB: serum | 8/ high |
| (Babadi et al., 2024) | The MWHS Study | Baltimore, Maryland, USA | Longitudinal Cohort Study | Pre- and perimenopausal women aged 45–54 years with intact uteri and both ovaries | 751 | Phthalate metabolites: urine | E2, T, P: serum | 8/ high |
| (Hoffmann-Dishon et al., 2024) | Unnamed Survey | Israel | Prospective Cohort Study | Women aged 19 to 38 years, undergoing a first to fifth IVF treatment | 72 | 12 phthalate metabolites, 12 phenolic substances: follicular fluid | E2, PG, AMH, INHB: follicular fluid | 7/ medium |
| (Warner et al., 2007) | The SWHS Study | Seveso, Italy | Prospective Cohort Study | Women exposed to TCDD during the 1976 Seveso, Italy | 363 | TCDD: serum | E2, P: serum Ovarian cysts, Ovarian follicles, Ovulation rate: transvaginal ultrasound | 8/ high |
| (Wieczorek et al., 2024) | Unnamed Survey | Poland | Prospective Cohort Study | Women of reproductive age (25-39 years) attending an infertility clinic. | 511 | The air pollutants concentrations were from the National Environmental Protection Inspectorate | AFC: ultrasonography AMH, FSH, E2: serum | 8/ high |
| (Kim et al., 2021) | Unnamed Survey | Seoul, South Korea | Prospective Cohort Study | Infertile women | 2276 | The air pollutants concentrations data were from the National Institute of Environmental Research | AMH: Serum | 9/ high |
| (Pang et al., 2023) | Unnamed Survey | Shandong Province, China | Retrospective Cohort Study | Women residents in Shandong Province | 18878 | The air pollutants concentrations data were collected from monitoring sites of the China National Environmental Monitoring Center and the China Atmosphere Watch Network. Reanalysis data on O_3_ column amount were obtained from the National Aeronautics and Space Administration website. | AMH: Serum | 8/ high |
| (Namvar et al., 2023) | The TLGS Study | district 13 of Tehran, Iran | Prospective Cohort Study | Women aged 20-50 years, living in district 13 of Tehran | 806 | The average annual exposure of people to air pollutants such as PM2.5, PM10, NO, NO_2_, NO_X_, SO_2_, and BTEX were estimated using previously developed land use regression models | AMH: serum | 9/ high |
| (Mok-Lin et al., 2010) | Unnamed Survey | USA | Prospective Cohort Study | Female partners of couples seeking infertility evaluation and treatment | 84 | BPA: urine | FSH, E2: Serum  The number of oocytes: be identified by the embryologists | 8/ high |
| (Liu et al., 2023) | Unnamed Survey | Hubei, China | Retrospective Cohort Study | Chinese Women of reproductive age with a focus on ovarian reserve measured by AMH levels | 5189 | The gridded concentrations of PM2.5 and its major constituents were collected from the Tracking Air Pollution in China database | AMH: Serum | 8/ high |
| (Jurewicz et al., 2019) | Unnamed Survey | Poland | Prospective Cohort Study | Female aged 25–39 years who attended the infertility clinic for diagnostic purposes | 511 | TCS: urine | AFC: transvaginal ultrasound AMH, FSH, E2: serum | 6/ medium |
| (LaPointe et al., 2024) | Unnamed Survey | Atlanta, Georgia, USA | Retrospective Cohort Study | Young, healthy oocyte donors in a large metropolitan city in the southeastern United States | 472 | Daily ambient PM2.5, NO_X_, and CO exposure data at a 250 m spatial resolution for 2008–2019 were generated using research line-source dispersion model for near-surface releases combined with regional air quality modeling and data fusion | AFC: transvaginal ultrasonography | 8/ high |
| (La Marca et al., 2020) | Unnamed Survey | Italy | Retrospective Cohort Study | All the laboratory AMH measurements performed at the Central Laboratory of the Ospedale Civile of Baggiovara | 1463 | Daily PM and NO_2_ exposure were registered through the monitoring network of the quality of the air for the province of Modena | AMH: serum | 5/ medium |
| (Kim et al., 2021) | Unnamed Survey | South Korea | Prospective Cohort Study | Couples undergoing IVF treatment | 146 | BPA: urine, plasma, follicular fluid | peak E2 level: Serum | 9/ high |
| (Parikh et al., 2024) | Unnamed Survey | India | Prospective Cohort Study | Indian women undergoing ICSI | 245 | Phthalate metabolites: follicular fluid | AMH levels, peak E2 levels: serum AFC, total number of oocytes, MII stage oocytes: transvaginal ultrasonography | 8/ high |
| (Kido et al., 2014) | Unnamed Survey | Vietnam | Prospective Cohort Study | 51 lactating women from the hot-spot area and 58 from the non-exposed area | 109 | Dioxin: breast milk | Cortisol, Cortisone, Dehydroepiandrosterone, Androstenedione, estrone, and E2 levels: serum, saliva | 6/ medium |
| (Whitworth et al., 2015) | Unnamed Survey | limpopo, South Africa | Prospective Cohort Study | Women from limpopo, South Africa studied in 2010-2011 | 420 | DDT, DDE: plasma | AMH: serum | 6/ medium |
| (Yao et al., 2024) | Unnamed Survey | Wuhan, China | Prospective Cohort Study | Women undergoing IVF | 525 | Phthalate metabolites: urine | AFC: transvaginal ultrasonography | 8/ high |
| (Kim et al., 2021) | The Biocycle Study | USA | Prospective Cohort Study | Healthy women of childbearing age | 251 | Cd: serum | AMH: serum | 7/ medium |
| (Jackson et al., 2011) |  | USA | Prospective Cohort Study | Healthy premenopausal women had a self-reported cycle length between 21 and 35 days | 252 | Cd, Pb, Hg: serum | FSH, LH, E2: serum | 9/ high |
| (Wright et al., 2015) | Unnamed Survey | Massachusetts, USA | Prospective Cohort Study | Women undergoing IVF | 205 | Hg: hair | FSH, E2: serum | 9/ high |
| (Gregoire et al., 2021) | The Sister Study | USA | Prospective Cohort Study | Premenopausal women | 883 | The air pollutants concentrations data were collected from the air quality system, interagency monitoring of protected visual environments networks, and the ozone monitoring instrument | AMH: serum | 6/ medium |
| (Liu et al., 2024) | Unnamed Survey | Hubei, China | Retrospective Cohort Study | Women who attended a fertility center | 4544 | O_3_: Tracking Air Pollution in China database | AMH: serum | 7/ medium |
| (Merklinger-Gruchala et al., 2022) | Unnamed Survey | Krakow, Poland | Prospective Cohort Study | 132 healthy, urban women | 132 | PM10: municipal ecological monitoring data | E2, P: saliva | 8/ high |
| (Wang et al., 2024) | Unnamed Survey | Tianjin, China | Prospective Cohort Study | Women seeking infertility treatment | 2212 | Daily mean outdoor PM2.5 and O_3_ concentrations were obtained from the Tracking Air Pollution in China dataset | AMH: serum | 9/ high |
