# Supplementary material for: Assessment of ovarian dysfunction induced by environmental toxins: a systematic review
Source: Front Public Health. 2025 Jul 30;13:1575418. doi: 10.3389/fpubh.2025.1575418 (PMC12343636; doi:10.3389/fpubh.2025.1575418)
Supplement: Supplementary file 4 [file Table_4.docx]

Supplementary Table A.4 The table of study population characteristics

| **Author (Year)** | **Specific population, condition, domain or measured symptom being studied** | **Mean age  (or Age range)** | **Gender/sex** | **Nation/Region** | **Types of exposed pollutants** | **Sample detect** | **Other relevant sociodemographics** |
| --- | --- | --- | --- | --- | --- | --- | --- |
| Emily S Barrett(2015) | General Population: only women | 30.7 | Female | Tromsø, Norway | Perfluoroalkyl substances (PFASs): PFOS, PFOA, PFOSA, PFDA | PFASs: serum | Subjects included in the current analysis 63% were married. Approximately half (49%) of subjects were parous. Most (79%) were nonsmokers and a majority (94%) reported at least some alcohol use. In bivariate analyses, parous women were slightly older than nulliparous women, with a slightly higher BMI. Parous women were more likely to smoke and less likely to engage in intense physical activity. |
| Astrid L. Beck(2024) | Infertile couples undergoing in vitro fertilization | 38 | Couples | Denmark | 16 Phthalates, 3 Phthalate substitutes, 6 Benzophenones | follicular fluid | The women went through a total of 155 treatments, hereunder 78 women (70 %) went through only one IVF or ICSI treatment, and 33 women (30 %) went through two to four treatments. Other factor female infertility constituted the largest proportion of diagnoses behind fertility treatment (55 %), whilst male factor infertility composed the second largest (17 %). Out of the 155 treatments, 40 cases of heartbeat at gestational week 7 was observed of which 33 resulted in a live birth. The median age of the participating women was 38 years, whilst for their male partners it was 37 years. Both women and men were generally younger if they subsequently obtained a live birth compared to those who did not (p < 0.01 and p = 0.03, respectively). |
| Richelle D. Björvang(2022) | Women undergoing assisted reproductive technology | 21-43 | Female | Uppsala, Sweden | 9 Organochlorine Pesticides (OCPs), 10 Polychlorinated Biphenyls (PCBs), 3 polybrominated diphenyl ethers (PBDEs), and 8 Perfluoroalkyl substances (PFASs) | OCPs, PCBs, PBDEs, and PFASs: serum, follicular fluid | They were mostly nulliparous, had a median BMI of 23 kg/m2. The cause of infertility was 29.2% female factor, 23.8% male factor, 7.5% both male and female, and 39.5% unexplained. The table shows demographic, lifestyle, and reproductive characteristics of the cohort at the time of recruitment. Fertilization rate was 66.7% with an average embryo score of 7.3 and 73% having at least one top-quality embryo. There were 157 fresh transfers, resulting in 66 clinical pregnancies (42%) and 59 live births (38%). There were 90 frozen transfers (range 0–4 frozen transfers per woman), resulting in 39 clinical pregnancies (43%) and 30 live births (33%). |
| Michael S Bloom(2011) | Women undergoing IVF | 28-44 | Female | San Francisco, USA | BPA | BPA: serum | At the time of oocyte retrieval, women were a mean (±SD) 35.8 (±4.1) years of age. Most were never-smokers (84.1%) and a substantial proportion Asian (29.6%). Median serum BPA concentration was 2.5 ng/mL (range = 0.0–67.4 ng/mL); a majority exceeded the limit of detection (86.4%). Peak E2 reached a median of 2167.0 pg/mL serum during ovarian stimulation, and a median of 10.5 oocytes (range = 2–35) were retrieved from each woman. Before ovarian stimulation, a median of 10.0 baseline antral follicles (range = 2–29) were counted for each woman, and just before the hCG trigger a median 9.0 mature-sized follicles (range = 2–25) were visualized. Basal FSH was a median 6.4 IU/L serum (range = 0.0–12.5). Approximately 36.4% of the infertility diagnoses were unexplained infertility, whereas 20.5% carried the diagnosis with diminished ovarian reserve, and an additional 20.5% were male infertility diagnoses. |
| Natalie M Crawford (2017) | Women without infertility | 33.3 | Female | Raleigh, Durham, and Chapel Hill, North Carolina area, USA | PFCs (including PFOA, PFOS, Perfluorononanoic acid (PFNA), and (PFHxS.) | PFCs: serum | Participants tended to be parous (60%), Caucasian (87%), highly educated (64% with at least some graduate degree work), and with a normal body mass index (62% between 18.5 and 24.9; calculated as weight (kg)/[height (m)]). Sixty-seven percent of women in the study became pregnant, with 12% of women conceiving in the first observed cycle. Women in the highest quartile of sum PFC exposure were overall similar to women with lower exposure levels. However, women in the highest exposure group were more likely to have longer mean cycle lengths (30.7 versus 28.7 days, p = 0.02) and less likely to achieve pregnancy at study end (54% versus 75%, p = 0.04) as compared to women with lower sum PFC exposures. |
| Ning Ding (2022) | Premenopausal women | 47.0–50.8 | Female | USA | 9 PFAS homologs, including perfluorohexane sulfonate (PFHxS), linear-chain PFOS (n-PFOS), the sum of branched-chain PFOS (Sm-PFOS), linear-chain PFOA (n-PFOA), the sum of branched-chain PFOA (Sb-PFOA), PFNA, perfluorodecanoate, perfluoroundecanoate, and perfluorododecanoate | PFAS: serum | A total of 577 women (51.5%) were White, 235 (21.0%) were Black, 142 (12.7%) were Chinese, and 166 (14.8%) were Japanese. More than half had received a college education and had never smoked. Most participants had given birth to at least 1 child, and 22.1% had a history of prior hormone use. The median physical activity score was 7.9 (interquartile range, 6.6–9.0), indicating moderate physical activity. The median body mass index was 26.1 (interquartile range, 22.7–31.5). |
| Michael S. Bloom (2017) | Female patients undergoing a 1st completed IVF cycle | 28-42 | Female | San Francisco, USA | 43 Polychlorinated Biphenyl (PCB) congeners, p,p'-dichlorodiphenyltrichloroethane (DDT), and its persistent metabolite p,p'-dichlorodiphenyldichloroethylene (DDE) | PCB, DDT, DDE: follicular fluid | Women were 28–42 years of age at the time of the procedure and with substantial proportions of Asians (n = 8, 25%) and former or current cigarette smokers (n = 6, 18.8%). Most IVF procedures used ICSI (75%). We retrieved a total of 424 oocytes from n = 32 study participants, 362 (85.4%) in M2-arrest and 216 (50.9%) of which fertilized normally. A total of 208 zygotes were subsequently assessed for embryo quality, 89 (42.8%) of which were graded as ‘good.' There were n = 14 (43.8%) implantations and n = 9 (28.1%) live births. |
| Lanlan Fang (2023) | Female partners from the longitudinal assisted reproduction cohort in Anhui | 31.53 | Female | Anhui, China | PM2.5, PM10, SO2, CO, NO2,O3 | PM2.5, PM10, SO2, CO, NO2,O3: collected from China High Air Pollutants | For all observations, the mean values of BMI at the point of reproductive hormone testing was 22.29 kg/m2. 38.74% were in middle school and lower, 38.25% were in high/technical school, and 23.00% were in college or above. 63.02% were female factor, and 36.98% were non-female factor. 41.62% resided in rural areas and 58.38% in urban areas. |
| Gaskins, Audrey J. (2019) | 632 women presenting to the Massachusetts General Hospital Fertility Center for infertility treatment | 18–45 | Female | USA | PM2.5 | PM2.5: estimated with a validated hybrid model of satellite-derived aerosol optical depth measurements and land-use terms | The 632 women had a mean (SD) BMI of 24.4 (4.7) kg/m2. The majority of women were never smokers (73%) and of Caucasian race (84%) with a college degree or higher (93%). The most common infertility diagnosis at enrollment was unexplained (41%). Women in our cohort resided in Massachusetts (96%), New Hampshire (2%), and Rhode Island (1%), as well as Maine and a few states outside of New England (<1%). |
| Xiaoqin Feng (2021) | 600 women from January 2013 to December 2019 in Reproductive Medical Center of Shanxi Provincial People’s Hospital in Shanxi of China | 30.58 | Female | Shanxi, China | SO2, NO2, PM10, PM2.5, CO, O3 | SO2, NO2, PM10, PM2.5, CO, O3: estimated based on the air pollutants monitoring data in the eleven prefectural-level cities | The average BMI of women in this study was 22.74 kg/m2 (SD = 3.49). Most participants were employed (75.7%), no university degree (64.5%), no parity (67.8%), no smoking (97.3%), and had regular pattern of menstrual cycles (92.7%). Infertility diagnosis included female factor (63.7%), male factor (14.3%) and unexplained infertility (22.0%). The duration of infertility lasted <2 years (25%), 2–5 years (57.7%) and >5 years (17.3%). |
| Robert B. Hood (2021) | Women, attending the Massachusetts General Hospital Fertility Center for infertility evaluation and treatment | 18-45 | Female | USA | PM2.5 | PM2.5: used a validated hybrid model of satellite and land use data with a 1 km2 spatial resolution | Race, n (%) White 477 (84.6%) Black 17 (3.0%) Asian 47 (8.3%)  BMI, kg/m2 24.5 (4.6%)   Smoking status, n (%) Never smoked 416 (73.6%) Ever smoked 149 (26.4%)  Education, n (%) < College 43 (7.65%) College graduate 241 (42.7%) Graduate degree 281 (49.7%)  Total physical activity (hr/week) 6.5 (8.2%)  History of being pregnant, n (%) 250 (44.3)  Initial infertility diagnosis, n (%) Male factor 153 (27.1%) Female factor 187 (33.1%) |
| Yao-Yao Du (2018) | Women aged between 20 and 45 years, with indications for IVF or ICSI | 27-35 | Female | Wuhan, China | Phthalate metabolites (including monomethyl Phthalate (MMP), monoethyl Phthalate (MEP), mono-n-butyl Phthalate (MBP), monobenzyl Phthalate (MBzP), MEHP, mono(2-ethyl-5-hydroxyhexyl) Phthalate (MEHHP), mono(2-ethyl-5-oxohexyl) Phthalate (MEOHP) and mono-n-octyl Phthalate (MOP)) | Phthalate metabolites: urine | The present study comprised 415 women with an average BMI of 21.5 kg/m2. Most of them were Han (96.9%) and non-smokers (94.9%). A total of 229 women (55.2%) were nulliparous. Nearly half of the subjects underwent IVF or ICSI due to tubal or pelvic infertility (40.0%), followed by male factor (23.9%) and diminished ovarian reserve (13.5%). |
| Ryan S. Babadi (2024) | Pre- and perimenopausal women aged 45–54 years with intact uteri and both ovaries | 45-54 | Female | Baltimore, Maryland, USA | Phthalate metabolites (including monoethyl Phthalate (MEP), MBP, MiBP, mono-(3-carboxypropyl) Phthalate (MCPP), monobenzyl Phthalate (MBzP), mono-2-ethylhexyl Phthalate (MEHP), mono-(2-ethyl-5-hydroxyhexyl) Phthalate (MEHHP), mono-(2-ethyl-5-oxohexyl) Phthalate (MEOHP), and mono-(2-ethyl-5-carboxypentyl) Phthalate (MECPP)) | Phthalate metabolites: urine | Most women were non-Hispanic White (66%), employed (80%), college-educated or higher (65%), married/partnered (65%), premenopausal (64%), drank alcohol (average ≥ 1 drink/month) (66%), never smoked (55%), and reported medication/supplement use (57%). Baseline AMH concentrations (GM (GSD)) were 0.30 (2.8) ng/mL. |
| Nathalie Hoffmann-Dishon (2024) | Women aged 19 to 38 years, undergoing a first to fifth IVF treatment due to male factor, unexplained infertility, or preimplantation genetic testing for monogenic disorders of autosomal recessive diseases | 30.9 | Female | Israel | 12 Phthalate metabolites; 12 Phenolic substances | 12 Phthalate metabolites, 12 phenolic substances: follicular fluid | FF samples from 72 women with a mean BMI of 23.1 ± 4.4 kg/m2. About two-thirds of the participants (63.8%) underwent their first IVF cycle at the time of FF collection. Thirty women underwent PGT-M. |
| Kristen W Smith (2013) | Women undergoing fertility treatment and their ovarian reserve markers in relation to urinary paraben concentrations. | 21.0–46.7 | Female | Boston, Massachusetts, USA | Parabens (methylparaben, Propylparaben, Butylparaben) | Parabens: urine | Women were primarily Caucasian, non-smokers and had a mean BMI (± SD) of 25.4 ± 5.15). The Society for Assisted Reproductive Technology diagnosis was most commonly female factor, followed by male factor and unexplained infertility. There was no significant difference in age or the number of participants diagnosed with PCOS in each of the three outcome subgroups. |
| Marcella Warner (2007) | Women exposed to TCDD during the 1976 Seveso, Italy, explosion | 31.3 | Female | Seveso, Italy | 2,3,7,8-Tetrachlorodibenzo-p-dioxin (TCDD) | TCDD: Serum | All women were Caucasian, 75% had finished more than the required amount of education, 67% had ever married, 17% were overweight or obese (BMI > 25 kg/m2), 58% had ever used oral contraceptives, 61% had never smoked, 88% currently drank coffee, 53% were parous, and 46% had reached menarche before the explosion. Overall, the median lipid-adjusted serum TCDD level for the 363 women was 77.3 ppt (interquartile range, 33–214 ppt; range, 2.8–17,300 ppt) |
| Katarzyna Wieczorek (2024) | Women of reproductive age (25-39 years) attending an infertility clinic. | 33.3 | Female | Poland | Sulfur dioxide (SO2), nitrogen dioxide (NO2), carbon monoxide (CO), ozone (O3), particulate matter (PM2.5 and PM10) | The air pollutants concentrations were obtain the National Environmental Protection Inspectorate | The majority of participants had higher (75.34%, n = 385) or secondary (21.14%, n = 108) education, the mean BMI (body mass index) 23.18 ± 3.80 kg/m2. The women were mostly nonsmokers (92.17%, n = 471), and 55% (281) of study subjects announced that they do not drink alcohol or drink less than 1 drink per week whereas 224 (44%) declare drinking 1–3 drinks per week. The initial diagnosis of infertility during recruitment was: male factor (37.8%, n = 193), idiopathic infertility (31.1%, n = 159), endometriosis (13.7%, n = 70), ovarian factor (4.7%, n = 24) and tubal factor (10.2%, n = 52). The duration of couple's infertility declared by study participant were: over 5 years (35.23%, n = 180), 3–5 years (29.55%, n = 151), 2–3 years (27.59%, n = 141) and 1–2 years (7.63%, n = 39). |
| Hannah Kim (2021) | Infertile women/Air pollution and ovarian reserve/Reproductive health/AMH | 36.6 | Female | Seoul, South Korea | PM10, PM2.5, NO2, CO, SO2, O3 | The air pollutants concentrations data were from the National Institute of Environmental Research | The 2276 women were working at the time of the ovarian reserve test (62.9%), had normal weight (62.8%), and reported no history of smoking (97.8%). The AMH level was 3.3 (3.1) ng/mL in total population. Average AMH ratio was 0.8 (0.7) and AMH < 0.5 ng/mL was observed in 10.3% (n=235) of total population and 8.6% (n=81) of Seoul residents. |
| Lihong Pang (2023)​ | 32,341 women residents in Shandong Province, who had received serum AMH measurements without controlled ovarian stimulation | 31.6 | Female | Shandong Province, China | PM1,PM2.5,PM10,NO2,O3 | The daily data on PMs, NO2 and max 8-h average O3 is collected from monitoring sites of the China National Environmental Monitoring Center and the China Atmosphere Watch Network. Reanalysis data on O3 column amount were obtained from the National Aeronautics and Space Administration (NASA) website. | More than half of the participants were nulliparae aged ≥30 years, with BMI <24 kg/m2, living in inland areas, and with regular menstruation. |
| Lidia Mínguez-Alarcón (2021) | Women attending a fertility center, focusing on ovarian reserve measured by AFC​ | 35 | Female | USA | Hg | Hg: hair | Women were predominantly Caucasian (84%). Their median (IQR) BMI was 23.1 (21.4, 26.1) kg/m2 and 74% had never smoked. Around half of the women (45%) had undergone a previous fertility evaluation and 38% had been previously pregnant. |
| Zahra Namvar (2023) | Women aged 20-50 years, living in district 13 of Tehran | 43 | Female | district 13 of Tehran, Iran | PM10, PM2.5, SO2, NO, NO2, NOX, benzene, toluene, ethylbenzene, p-xylene, m-xylene, o-xylene (BTEX), and total BTEX | The average annual exposure of people to air pollutants such as PM2.5, PM10, NO, NO2, NOX, SO2, and BTEX compounds (benzene, ethylbenzene, toluene, p-xylene, m-xylene, o-xylene, and total BTEX) were estimated using previously developed land use regression (LUR) models. | The median (IQR) BMI of participants was 28.18 (25.29–31.24) kg/m2, the majority of members were married, had 6–12 years of education, and had low physical activity. |
| E Mok-Lin (2010) | Female partners of couples seeking infertility evaluation and treatment | 35.6 | Female | USA | Bisphenol A (BPA) | BPA: Urine | Seventy-four (88%) women were Caucasian. BMI ranged from 16.5 to 42 kg/m2, with a mean ±SD of 24.0 ± 5.1. Sixty (71%) women had never smoked and three women were current smokers. A majority of the subjects had SART diagnoses of female factor (35%) or male factor infertility (36%); unexplained infertility was present in 29% of the subjects. Day 3 FSH levels ranged from 1.0 to 15.2 IU/L, with a mean ± SD of 7.7 ± 2.3. |
| Lidia Mínguez-Alarcón (2017) | Women seeking care at a fertility center | 36 | Female | USA | Triclosan | Triclosan: Urine | This analysis included 109 women with BMI of 23.0 kg/m2 (IQR 20.9, 26.5). The majority of the participants were Caucasian (78%), with a college degree or higher (80%), and most had never smoked (73%). Most of the women (78%) had undergone infertility evaluation, and 37% had been treated for infertility before their enrollment in the study. Unexplained infertility was the primary infertility diagnosis at enrollment (50%). |
| Carmen Messerlian (2016) | Women seeking infertility investigation and treatment | 35.7 | Female | USA | Phthalate metabolites | Phthalate metabolites: Urine | The study cohort comprised 215 women, predominantly Caucasian (81%) and never-smokers (74%), with an average age of 35.7 years at time of enrollment. Most women were nulliparous (88%), and 42% had a female factor as the primary cause of infertility. |
| Shuangyan Liu (2023) | Chinese Women of reproductive age with a focus on ovarian reserve measured by AMH levels | 32.3 | Female | Hubei, China | PM2.5, Black Carbon (BC), Ammonium (NH4+), Nitrate (NO3-), Organic Matter (OM), Sulfate (SO4^2-) | The dataset of PM2.5 constituents was established by combining ground observations, the Weather Research and Forecasting-CMAQ modeling system, a machine learning algorithm, and PM2.5 data merged from multiple sources, with the correlation coefficients of 0.64–0.75 | The mean BMI (± SD) were and 22.0 ± 3.0 kg/m2, respectively. Overall, 50.5 % were employed, 36.6 % had university degrees, 0.3 % smoked and drank, 16.6 % exposed to second-hand smoking, and 45.5 % lived in urban areas. Female factors of infertility including endometriosis, tubal factor, DOR, ovulation disorders, and uterine disorders contributed the most (74.5 %) to the infertility factors, with the mean of duration of infertility (± SD) of 3.4 ± 2.7 years, and 20.5 % women were multiparous. The mean of AMH levels (± SD) was 3.0 ± 2.2 ng/mL. |
| Joanna Jurewicz (2019) | Five hundred eleven female aged 25–39 years who attended the infertility clinic for diagnostic purposes | 33.3 | Female | Poland | TCS | TCS: Urine | Most of women had higher (75.34%) or secondary (21.14%) education and were non smokers (92.17%). The mean (±SD) body mass index (BMI) were and 23.18 ± 3.80 kg/m2 respectively. Most of the study female drank none or less than 1 drink per week (55.0%). Duration of couple’s infertility lasted mostly > 5 years (35.23%) and 3–5 years (29.55%). The initial infertility diagnosis was mostly male factor (37.8%), followed by idiopathic infertility (31.1%) and female factor (28.6%) |
| Sarah LaPointe (2024) | Young, healthy oocyte donors in a large metropolitan city in the southeastern United States | 25 | Female | Atlanta, Georgia, USA | Nitric oxide (NOx), CO, PM2.5 | Daily ambient PM2.5, NOx, and CO exposure data at a 250 m spatial resolution for 2008–2019 were generated using research line-source (R-LINE) dispersion model for near-surface releases combined with regional air quality modeling and data fusion | The median age of donors at oocyte retrieval was 25 years (range: 21 to 32 years), 70% were non-Hispanic White, and 55% had completed at least a four-year college degree (Table 1). The majority of donors were never smokers (90%), had a BMI < 25 kg/m2 (81%), and were nulliparous (75%). Across the census-tracts our donors resided in, the median family income was $60,438 (range $15,682 to $178,438), the median home value was $184,550 (range $57,500 to $882,800), and the median percentage of families below poverty was 12% (range < 1 to 54%). |
| Antonio La Marca (2020) | The laboratory AMH measurements performed at the Central Laboratory of the Ospedale Civile of Baggiovara without any clinical information about patients | 38.8 | Female | Italy | PM10, PM2.5, NO2 | Daily particulate matter (PM) and NO2 exposure were registered through the monitoring network of the quality of the air for the province of Modena | None reported |
| Hyun-Ki Kim (2019) | Couples undergoing IVF treatment | Women: 34.8 Men: 36.3 | Male and female | South Korea | Bisphenol A (BPA) | BPA: urine, plasma, follicular fluid and semen | Ethnicity: 48 Asian (47 Korean and 1 Japanese), 98 Russian couples; Female BMI: 21.6 (20.2, 24.0)kg/m;  No embryos transferred: 9 (6.2%)  1 embryo: 21 (14.4%) 2 embryos: 77 (52.7%) 3 embryos: 39 (26.7%) |
| Firuza Rajesh Parikh (2023) | Indian women undergoing intracytoplasmic sperm injection (ICSI) | 34 | Female | India | Phthalate metabolites: mono-n-butyl Phthalate (MBP), mono-ethyl Phthalate (MEP), mono-isononyl Phthalate (MiNP), mono-isodecyl Phthalate (MiDP), mono(2-ethyl-5-oxohexyl) Phthalate, and mono(2-ethyl-5-hydroxyhexyl) Phthalate | Phthalate metabolites: Follicular fluid | BMI |
| Teruhiko Kido (2014) | 51 lactating women from the hot-spot area and 58 from the non-exposed area | 20-30 | Female | Vietnam | Dioxin (PCDDs+PCDFs) | Dioxin: breast milk | Hot-spot area  (n = 51) Weight (kg) 48.5 ± 6.63 Hight (cm) 152.3 ± 5.47 BMI (kg/m2) 20.9 ± 2.22 Residence period (years)  21.0 (21.0-26.0) Family income (×104VND/month) 200 (100-300)  Non-exposed area (n = 58) Weight (kg) 48.8 ± 5.04 Hight (cm) 152.7 ± 5.10 BMI (kg/m2) 20.9 ± 1.90 Residence period (years)  22.5 (18.8-25.0) Family income (×104VND/month) 200 (110-300) |
| Whitworth, Kristina W. (2015) | 420 women from limpopo, South Africa studied in 2010-2011 | 20-30 | Female | Limpopo, South Africa | p,p′-DDT, p,p′-dichlorodiphenyldichlorethylene (DDE) | DDT, DDE: plasma | BMI (kg/m2): median (QR)  24.7 (21.5-28.3) DDT (μg/L): median (IQR) 0.89(0.27-2.7) DDE (μg/L): median (IQR)  24(22-26)  Married No 267 Yes/cohabitating 158  Family income (Rand) <1,250 106 1,250-1,999 106 2,000-3,000 109 >3,000 104  Education <Grade 11 100  Grade 11 121 Grade 12 131 >Grade 12 73  Indoor residual spraying None 194 DDT spraying 125 Pyrethroid spraying 88 |
| Yangcheng Yao (2024) | 525 women undergoing IVF | 31.1 | Female | Wuhan, China | Phthalate metabolites (mono-ethyl Phthalate (MEP), mono-methyl Phthalate, mono-n-butyl Phthalate (MBP), mono(2-ethylhexyl) Phthalate (MEHP), mono-benzyl Phthalate, mono(2-ethyl-5-oxohexyl) Phthalate (MEOHP), mono(2-ethyl-5-hydroxyhexyl) Phthalate (MEHHP) and mono-n-octyl Phthalate (MOP)) | Phthalate metabolites: urine | The average (± SD) BMI of the study population were 21.9 ± 2.7 kg/m², respectively, and most of them were of Han ethnicity (96.6%) and had never smoked (96.0%). The average (± SD) duration of infertility was 3.7 ± 2.9 years, and more than half of the subjects had primary infertility (54.5%) and were diagnosed with female factors (65.6%). The average (± SD) AFC was 13.4 ± 6.8. |
| Keewan Kim (2021) | Healthy women of childbearing age | 18-44 | Female | USA | Cd | Cd: serum | Women in the highest tertile of blood cadmium concentrations tended to be older (mean ± SD; highest tertile 29.9 ± 8.2 years of age, lowest tertile 25.7 ± 7.8) and self-identified as black or other than white or black for race/ethnicity. 4% of women were current smokers and smoking status was positively correlated with blood cadmium concentrations (P = 0.01). No other demographic or lifestyle characteristics were correlated with blood cadmium levels. |
| Diane L Wright  (2015) | Women undergoing IVF | 27-43 | Female | Massachusetts, USA | Hg | Hg: Hair | Most women were Caucasian (82%) and non-smoking (74%). Approximately a third of couples were categorized to 3 different primary SART diagnoses; female factor, male factor, or unexplained infertility. In a subgroup of patients (n=157), the median fish consumption was 1.5 serving per week. |
| L.W.Jackson (2011) | Healthy premenopausal women had a self-reported cycle length between 21 and 35 days | 18-44 | Female | USA | Cd, Pb, Hg | Cd, Pb, Hg: serum | The study population was young with 51% of the women between the ages of 18 and 24 years (Table I). The majority of the women identified themselves as being white, non-Hispanic (59%) and 40% had a college degree or higher. Only 25% of the popu lation reported drinking alcohol at least once a week, and 81% reported never smoking. Sixty-nine percent of women reported never being pregnant; among the 76 women reporting a previous pregnancy, 65 women reported a previous live birth. Overall, 36% of the study population was overweight or obese |
| Gregoire, A. M.(2021) | Premenopausal women | 47 | Female | USA | Particulate matter (PM2.5, PM10) and nitrogen dioxide (NO2) | The air pollutants concentrations data were collected from the air quality system, interagency monitoring of protected visual environments networks, and the ozone monitoring instrument | Women tended to be highly educated (58.2% with at least a bachelor’s degree), non-Hispanic White (87.1%) and nonsmokers (91.7% never or past smoker) . The average concentration and IQR of pollutants at the study participants’ enrollment residences were 10.62 µg/m3 (IQR = 3.5 µg/m3), 22.26 µg/m3 (IQR = 5.4 µg/m3), and 10.48 ppb (IQR = 6.1 ppb) for PM2.5, PM10, and NO2, respectively. Most women (64.9%) lived more than 200 m from the nearest major road at enrollment. |
| Liu, S.(2024) | Women who attended a fertility center | 31.4 | Female | Hubei, China | Ozone (O3) | O3: Tracking Air Pollution in China database | the mean (SD) age and BMI were 31.4 (4.6) years and 22.4 (3.3) kg/m2, respectively. Overall, the majority were Han (95.9%), 29.2% had university degrees, 43.8% lived in rural areas, 0.7% smoked, 0.2% drank and 83.3% worked currently. Female factors contributed 81.8% to the infertility factors, with the mean (SD) duration of infertility of 3.4 (2.8). In addition, most women were nulliparous (84.8%), fresh cycle of IVF (84.6%), and had regular patterns of menstrual cycles (92.0%). The mean (SD) of AMH levels was 3.5 (2.7) ng/mL |
| Merklinger-Gruchala A(2022) | 132 healthy, urban women | 29.5 | Female | Krakow, Poland | Particulate matter (PM10) and tobacco smoke. | PM10: municipal ecological monitoring data | Participants were urban women, with most having regular menstrual cycles and no fertility or chronic disorders. |
| Xinyan Wang (2023) | Women seeking infertility treatment | 33.18 | Female | Tianjin, China | PM2.5 and Ozone (O3) | Daily mean outdoor PM2.5 and O3 concentrations were obtained from the Tracking Air Pollution in China dataset | The mean BMI was 22.76 ± 3.12 kg/m2, and 950 (42.95%) women were overweight or obese (BMI ≥24 kg/m2). The mean infertility duration was 4.30 ± 2.94 years. |
