# Supplementary material for: Assessment of ovarian dysfunction induced by environmental toxins: a systematic review
Source: Front Public Health. 2025 Jul 30;13:1575418. doi: 10.3389/fpubh.2025.1575418 (PMC12343636; doi:10.3389/fpubh.2025.1575418)
Supplement: Supplementary file 5 [file Table_5.docx]

Supplementary Table A.5 The Table of NOS Results for Evaluating Study Quality

| **Author (Year)** | **Selection** | **Comparability** | **Outcome/ Exposure** | **Total** | **Quality*** |
| --- | --- | --- | --- | --- | --- |
| Emily S Barrett (2015) | 3 | 2 | 3 | 8 | high |
| Astrid L. Beck (2024) | 4 | 1 | 3 | 8 | high |
| Richelle D. Björvang (2022) | 3 | 2 | 2 | 7 | medium |
| Michael S Bloom (2011) | 3 | 2 | 2 | 7 | medium |
| Natalie M Crawford (2017) | 4 | 1 | 3 | 8 | high |
| Ning Ding (2022) | 4 | 2 | 2 | 8 | high |
| Michael S. Bloom (2017) | 3 | 1 | 2 | 6 | medium |
| Lanlan Fang (2023) | 3 | 1 | 3 | 7 | medium |
| Audrey J. Gaskins (2019) | 3 | 2 | 3 | 8 | high |
| Xiaoqin Feng (2021) | 3 | 2 | 2 | 7 | medium |
| Robert B. Hood (2021) | 3 | 2 | 3 | 8 | high |
| Yao-Yao Du (2018) | 3 | 2 | 3 | 8 | high |
| Ryan S. Babadi (2024) | 4 | 2 | 2 | 8 | high |
| Nathalie Hoffmann-Dishon (2024) | 3 | 1 | 3 | 7 | medium |
| Kristen W Smith (2013) | 4 | 1 | 3 | 8 | high |
| Marcella Warner (2007) | 4 | 1 | 3 | 8 | high |
| Katarzyna Wieczorek (2024) | 4 | 1 | 3 | 8 | high |
| Hannah Kim (2021) | 4 | 2 | 3 | 9 | high |
| Lihong Pang (2023)​ | 3 | 2 | 3 | 8 | high |
| Lidia Mínguez-Alarcón (2021) | 4 | 2 | 3 | 9 | high |
| Zahra Namvar (2023) | 4 | 2 | 3 | 9 | high |
| E Mok-Lin (2010) | 3 | 2 | 3 | 8 | high |
| Lidia Mínguez-Alarcón (2017) | 4 | 2 | 3 | 9 | high |
| Carmen Messerlian (2016) | 4 | 2 | 3 | 9 | high |
| Shuangyan Liu (2023) | 3 | 2 | 3 | 8 | high |
| Joanna Jurewicz (2019) | 3 | 1 | 2 | 6 | medium |
| Sarah LaPointe (2024) | 3 | 2 | 3 | 8 | high |
| Antonio La Marca (2020) | 3 | 0 | 2 | 5 | Medium |
| Hyun-Ki Kim (2019) | 4 | 2 | 3 | 9 | high |
| Firuza Rajesh Parikh (2023) | 4 | 1 | 3 | 8 | high |
| Teruhiko Kido (2014) | 3 | 0 | 3 | 6 | Medium |
| Kristina W Whitworth (2015) | 3 | 1 | 2 | 6 | Medium |
| Yangcheng Yao (2024) | 3 | 2 | 3 | 8 | high |
| L.W.Jackson (2011) | 4 | 2 | 3 | 9 | high |
| Diane L Wright  (2015) | 4 | 2 | 3 | 9 | high |
| Keewan Kim (2021) | 4 | 1 | 2 | 7 | Medium |
| A. M. Gregoire（2021） | 4 | 1 | 1 | 6 | medium |
| Shuangyan Liu (2024) | 3 | 2 | 2 | 7 | medium |
| Merklinger-Gruchala A（2022） | 4 | 1 | 3 | 8 | high |
| Xinyan Wang (2023) | 4 | 2 | 3 | 9 | high |
| Quality: High-quality studies were those with NOS scores of 8 or higher, medium-quality studies scored between 5 and 7, and low-quality studies scored below 5. | | | | | |
